# Integrative bulk and single-cell transcriptome analyses reveal integrated stress response-related biomarkers in periodontitis with experimental validation

**DOI:** 10.3389/fimmu.2025.1705047

**Published:** 2025-12-11

**Authors:** Linling Du, Jie Pan, Nan Xu, Feng Yan, Weiyi Tian, Qiyan Li

**Affiliations:** Center of Stomatology, First Peoples Hospital of Yunna Province, Kunming, Yunnan, China

**Keywords:** periodontitis, integrated stress response, biomarkers, single-cell sequencing analysis, RT-qPCR

## Abstract

**Background:**

This study aimed to investigate the role of integrated stress response (ISR)-related biomarkers in periodontitis (PD).

**Methods:**

Transcriptomic data related to PD were obtained from public databases. A bioinformatics approach combined with machine learning techniques was used to identify ISR-associated molecular markers involved in PD pathogenesis and to validate their expression patterns. Pathway enrichment analyses and immune landscape characterization were performed to elucidate the molecular mechanisms of these markers in PD progression. Single-cell RNA sequencing (scRNA-seq) was employed to resolve cellular heterogeneity and examine the expression patterns of candidate biomarkers. Reverse transcription-quantitative polymerase chain reaction (RT-qPCR) assays were conducted to validate the expression profiles.

**Results:**

BTG2, DERL3, FOS, HSPA13, and YOD1 were identified as potential PD biomarkers. Among them, BTG2, DERL3, FOS, and HSPA13 were co-enriched in the “osteoclast differentiation” pathway. DERL3 showed the strongest positive correlation with plasma cells and the strongest negative correlation with resting dendritic cells (|cor| > 0.3, P < 0.05). scRNA-seq analysis highlighted T cells as the key population. During T cell differentiation, BTG2 expression initially increased, then decreased, followed by a subsequent rise in the mid-to-late stages; DERL3 expression exhibited a transient increase before returning to baseline; and FOS expression increased gradually throughout the process. RT-qPCR results confirmed that the expression levels of BTG2, DERL3, FOS, and HSPA13 were significantly upregulated, while YOD1 expression was downregulated in the PD group (P < 0.05), which was consistent with the database-predicted patterns.

**Conclusion:**

This study integrated bulk and single-cell RNA-seq analyses to identify BTG2, DERL3, FOS, HSPA13, and YOD1 as PD biomarkers, with T cells as the central cell type, providing novel diagnostic insights for PD.

## 1 Introduction

Periodontitis (PD) is a chronic inflammatory disorder triggered by an imbalance in the oral microbiota, leading to the gradual destruction of periodontal tissues, including the gums, periodontal ligament, and alveolar bone, ultimately resulting in tooth loss. As the sixth most prevalent disease globally, PD affects 10%–15% of the population, with 538 million cases reported in 2019 (1–3). The host immune response to dysbiotic biofilms plays a pivotal role in periodontal tissue degradation (4), where microbial dysbiosis initiates an immune-inflammatory cascade. Simultaneously, bacterial infiltration interacts with immune dysregulation, accelerating tissue destruction (5). Advanced PD severely impacts oral health-related quality of life and is associated with systemic inflammatory diseases, such as diabetes, rheumatic diseases, and atherosclerosis (6). Throughout disease progression, miRNAs (e.g., miR-146a/b, miR-155) are secreted by tissue cells, such as CD56+ NK cells, CD4+ T cells, and CD8+ T cells, transported *via* exosomes into saliva, and contribute to inflammatory signaling and tissue damage (7). However, the molecular mechanisms driving periodontal destruction remain poorly understood (8), and specific pathogenic genes, cellular pathways, as well as reliable prediagnostic markers and therapeutic targets, have yet to be identified (9).

The integrated stress response (ISR),a conserved signaling pathway activated by diverse stressors such as proteostasis disruption and nutrient deprivation (10), operates through the coordinated regulation of four eIF2α kinases—PERK, GCN2, HRI, and PKR (11). Recent studies have revealed that ISR plays a complex and critical role in periodontal diseases. Activation of the PERK-eIF2α-ATF4 axis has been shown to exacerbate osteoblast differentiation (12), while GCN2 appears to mitigate oral inflammation and tissue destruction, suggesting a protective function in periodontitis (13). Notably, PKR has been demonstrated to mediate osteoclast activation and play a pivotal role in LPS-induced alveolar bone loss (14). Furthermore, ISR has been proven to drive M1 macrophage polarization and amplify inflammatory responses (15). However, the precise activation dynamics and functional network of ISR in periodontitis remain to be fully elucidated. Given its dual regulatory effects on inflammation and bone metabolism, targeted therapeutic interventions against the ISR pathway—whether administered alone or in combination with other treatments—hold significant potential as promising strategies for periodontitis management.

In recent years, machine learning approaches have been widely applied in biomedical research for feature selection and model construction with high-dimensional data, demonstrating strong potential in identifying key disease-related genes and biomarkers (16). Transcriptomic data are typically characterized by high dimensionality and substantial noise (17), which often lead to overfitting when analyzed using traditional statistical methods (18). To address this issue, machine learning techniques can be optimized through strategies such as cross-validation to mitigate overfitting and enhance model generalizability (19). Therefore, this study employed three machine learning methods—Least Absolute Shrinkage and Selection Operator (LASSO), Support Vector Machine-Recursive Feature Elimination (SVM-RFE), and the Boruta algorithm—for feature selection. LASSO regression introduces an L1 regularization penalty term that shrinks the coefficients of non-informative variables to zero during the construction of a classification model, thereby achieving feature selection and dimensionality reduction, particularly in datasets with multicollinearity (DOI: 10.1016/j.aej.2025.03.061). SVM-RFE is a backward elimination algorithm based on model weights. It iteratively constructs SVM models, ranks features according to their weights (e.g., coefficients), and removes the least important features to identify an optimal feature subset that maximizes classification accuracy (20). The Boruta algorithm is a wrapper method based on random forest, which creates shuffled shadow copies of the original features as references and uses statistical testing to compare the importance of actual features against these shadow attributes, thereby identifying all features significantly associated with the dependent variable (21).By integrating these three methods, it is possible to leverage their respective strengths and achieve complementary advantages: LASSO provides a robust preliminary dimensionality reduction, SVM-RFE optimizes the feature subset from the perspective of maximizing the classification margin, and the Boruta algorithm helps ensure that no potentially relevant features are overlooked. This multi-angle, multi-strategy integration is expected to overcome the limitations of individual methods and significantly enhance the reliability, stability, and interpretability of the selected biomarkers.

The advent of single-cell RNA sequencing (scRNA-seq) has revolutionized our ability to investigate cellular heterogeneity. Different cell types, particularly immune cells, exhibit distinct functional, pathway, and regulatory profiles, making the characterization of cell-type-specific gene expression patterns essential for identifying disease biomarkers and therapeutic targets (22). scRNA-seq enables the detection of intercellular genetic and proteomic variations while providing microbial genetic data at single-cell resolution, offering unprecedented insights into microenvironmental dynamics (23). In PD research, this technology allows for the cell-type-specific identification of molecular alterations, facilitating investigations into gene function within bone-immune cell populations (24). Key findings indicate that transcriptional reprogramming of osteoblasts, osteoclast precursors, and immune cell subsets drives bone resorption in PD (25). By integrating scRNA-seq with bulk transcriptomic data, it is possible to precisely delineate disease-specific cellular subpopulations and regulatory networks, uncovering novel therapeutic opportunities.

This study primarily aims to identify candidate genes associated with the ISR in PD using publicly available transcriptomic datasets. Through machine learning and bioinformatics analyses, such as GSEA, GSVA, and immune infiltration profiling, this study identified ISR-related biomarkers in PD and explored their roles in disease pathogenesis, providing new perspectives for treatment strategies.

## 2 Materials and methods

### 2.1 Data acquisition

**Figure 1:**
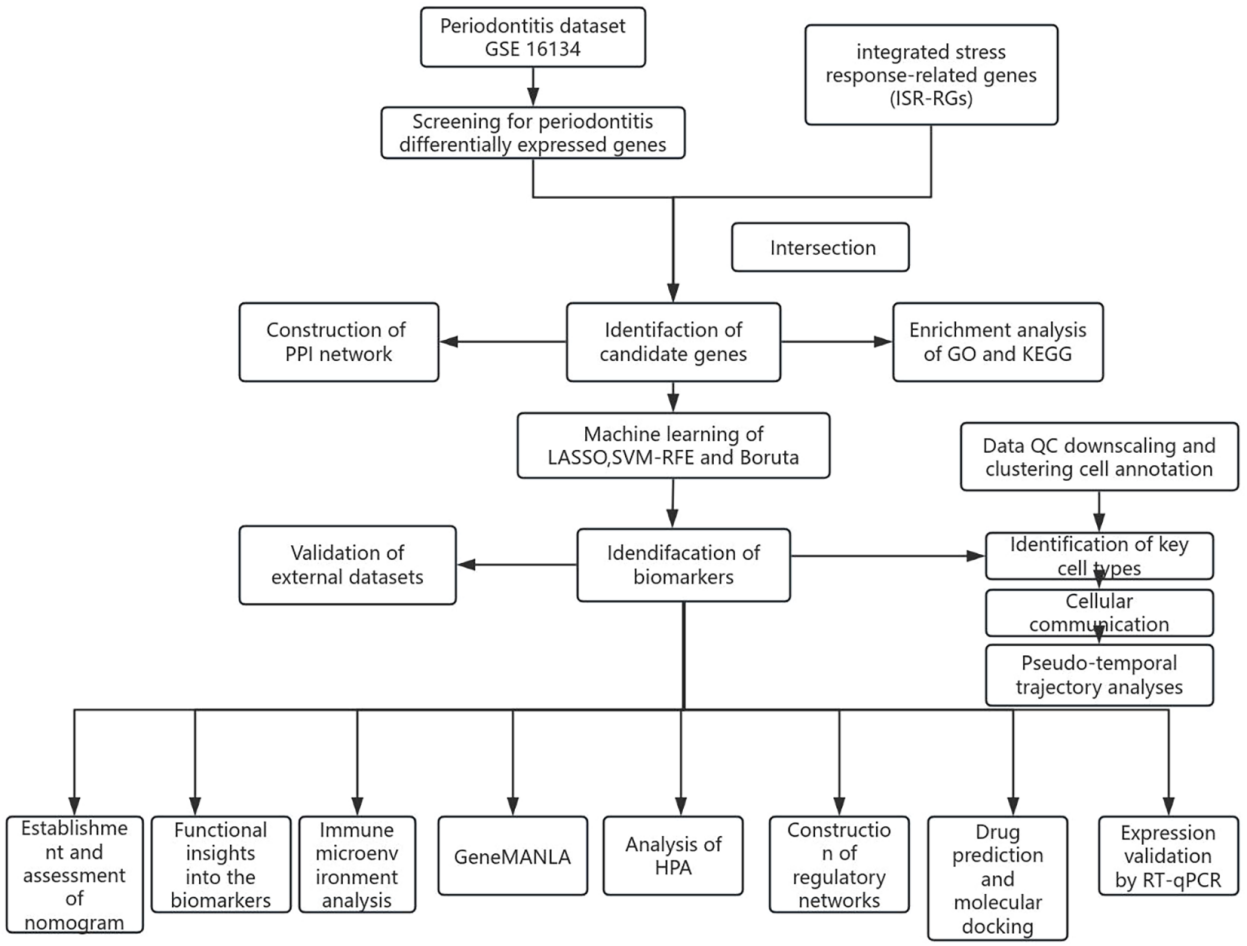
Analysis flowchart.

The PD datasets used in this study were sourced from the Gene Expression Omnibus (GEO) database (https://www.ncbi.nlm.nih.gov/geo/), specifically GSE16134 (platform: GPL570), GSE10334 (platform: GPL570), and GSE171213 (platform: GPL24676). The GSE16134 dataset was utilized as the training dataset. This dataset consisted of microarray data and included 310 samples (cases vs. controls = 241:69). These samples were obtained from 120 participants, with each participant contributing at least two interdental gingival papillae from the posterior maxilla. The data were used to identify differentially expressed genes (DEGs) and to construct a risk model. The GSE10334 dataset was used as the validation set, comprising 247 samples (183 cases and 64 controls) from 90 participants, investigating gene expression in both healthy and diseased gingival tissues. The single-cell RNA sequencing dataset (GSE171213), comprising periodontal tissue samples from 5 patients with periodontitis and 4 healthy controls, was used for single-cell analysis, while the single-cell transcriptomic dataset GSE164241 was employed for external validation. The GSE164241 dataset included samples from 13 healthy gingival mucosa tissues and 8 periodontitis gingiva tissues. Additionally, the ISR-RGs involved in this study were derived from (26). We systematically integrated multiple stress response-related gene sets covered in this literature, including the heat shock response (79 genes), oxidative stress response (58 genes), unfolded protein response (47 genes), hypoxia stress response (119 genes), and DNA damage response (231 genes). These gene sets were systematically compiled based on public databases (such as GO and Reactome) and experimental data from published literature. After merging all gene sets and removing duplicate genes, a final set of 529 unique ISR-RGs was obtained for subsequent analysis. The specific gene list is provided in **Supplementary Table S1**. The analysis workflow of this study is shown in **Figure 1**.

### 2.2 Analysis of differentially expressed genes

To identify genes with altered expression patterns, we performed data processing using the GSE16134 dataset with R software (version 4.3.2) and the Bioconductor platform, in which a comparative transcriptomic analysis was carried out between periodontitis patients and healthy controls. The limma statistical framework (version 3.58.1) (27) was applied to identify significantly dysregulated genes, with thresholds set at an absolute fold change >2 (|log_2_FC| > 1) and adjusted p-value <0.05. For data visualization, volcano plots were generated using the ggplot2 visualization toolkit (version 3.5.1) (28) to illustrate the distribution of differentially expressed genes (DEGs). Hierarchical clustering heatmaps were constructed using the ComplexHeatmap package (version 2.18.0) (29) to display expression patterns across samples.

### 2.3 Identification, enrichment analysis, and chromosomal and subcellular localization of candidate genes

The overlap between DEGs and ISR-RGs was analyzed using the ggvenn package (version 0.1.10) (30). This analysis revealed genes potentially associated with the ISR in PD, which were subsequently designated as candidate genes.

Following differential expression analysis, to elucidate the functions of the candidate genes, a systematic functional enrichment analysis was performed using the R package clusterProfiler (v4.10.1) (31) and the human annotation database org.Hs.eg.db. First, gene symbols were converted to Entrez IDs using the bitr() function to meet the data format requirements for subsequent analyses. Subsequently, Gene Ontology (GO) and Kyoto Encyclopedia of Genes and Genomes (KEGG) pathway enrichment analyses were conducted. For the GO enrichment analysis, the enrichGO() function was used with the following parameters: OrgDb = org.Hs.eg.db, ont = “ALL” (to include biological processes [BP], cellular components [CC], and molecular functions [MF]), and keyType = “ENTREZID”. To retain all information for independent filtering, the initial analysis set both pvalueCutoff and qvalueCutoff to 1, after which terms with p < 0.05 were selected as significant. For the KEGG pathway enrichment analysis, the enrichKEGG() function was applied (organism = “human”), and the setReadable() function was used to convert Entrez IDs back to more intuitive gene symbols in the results. Here, GeneRatio was defined as the ratio of the number of input genes assigned to a specific functional term or pathway to the total number of input genes, which was directly calculated from the clusterProfiler output. Similarly, KEGG pathways with p < 0.05 were considered significantly enriched. Finally, the significantly enriched results were visualized using the ggplot2 package.

To examine the chromosomal mapping and spatial organization of these genes, circular genomic visualization was employed using the RCircos tool (version 1.2.2) (32). Subcellular compartmentalization analysis was also conducted to gain a deeper mechanistic understanding of the candidate gene functions and their cellular roles. The subcellular localization of each candidate gene’s protein was predicted using the Hum-mPLoc 3.0 online platform (http://www.csbio.sjtu.edu.cn/bioinf/Hum-mPLoc3/).

### 2.4 Discernment of biomarkers through machine learning, receiver operating characteristic (ROC) analysis, and expression validation

Based on the candidate genes, three distinct machine learning methods were applied across the entire GSE16134 sample cohort, using the previously identified candidate gene set as input variables. Among these approaches, the least absolute shrinkage and selection operator (LASSO) regularization technique was employed for variable selection and dimensionality reduction, executed using the glmnet package (version 4.1.8) (33). At the optimal lambda parameter, the LASSO algorithm minimized error rates and identified genes with non-zero coefficients as signature features. For feature selection, the support vector machine-recursive feature elimination (SVM-RFE) method was utilized *via* the caret package (version 6.0.94) (34), employing 10-fold cross-validation. Variable optimization was guided by root mean square error (RMSE) metrics, with a decrease in RMSE reflecting improved predictive performance. The genes demonstrating the minimal RMSE were designated as SVM-RFE feature genes.

Following this, the genes identified through the minimal RMSE criterion were categorized as SVM-RFE signature genes. Additionally, the Boruta algorithm, implemented through the Boruta package (version 8.0.0) (35), was used to screen variables from the target gene pool. The importance score was calculated for both the true feature matrix and the shadow feature matrix, with the largest shadow feature importance score identified as shadowMax. True features displaying importance scores higher than shadowMax were classified as “important” and designated as Boruta feature genes. The VennDiagram package (version 1.7.3) (36) was then used to identify the overlapping signature genes identified by LASSO, SVM-RFE, and Boruta, facilitating the determination of common feature genes. Subsequently, ROC curves for the GSE16134 and GSE10334 datasets were generated using the pROC package (version 1.18.5) (37), evaluating the diagnostic performance of the common feature genes for PD through area under the curve (AUC) calculations. Specifically, shared signature genes exhibiting an AUC > 0.7 across both datasets were identified as potential biomarkers. Moreover, Wilcoxon rank-sum testing was applied to compare the expression profiles of these candidate biomarkers between PD and control groups in both the GSE16134 and GSE10334 datasets. Biomarkers exhibiting significant inter-group differences (P < 0.05) and displaying consistent expression trends in both datasets were defined as reliable biomarkers.

### 2.5 Establishment and assessment of the nomogram

A nomogram to predict the likelihood of PD development was constructed using the rms package (version 6.5.0) (38), based on biomarker data from the GSE16134 dataset. In this nomogram, each biomarker was assigned specific point values, with a higher total score correlating to an increased risk of PD.

To assess the predictive accuracy and clinical applicability of the nomogram, multiple validation methods were employed. Model calibration was evaluated using calibration plots generated by the calibrate function within the same framework (version 6.5.0), with P-values exceeding 0.05 indicating a good model fit. The discriminative ability of the nomogram was assessed using ROC analysis, and the AUC was calculated using the pROC package (version 1.18.5). To evaluate the clinical utility and net benefit of the predictive model in decision-making, decision curve analysis (DCA) was conducted using the ggDCA visualization package (version 1.2) (39).

### 2.6 Functional insights into the biomarkers

The GeneMANIA database (http://www.genemania.org/) was utilized to construct a gene-gene interaction (GGI) network, illustrating the relationships between the identified biomarkers and genes with similar functions, along with their shared biological roles. Gene set enrichment analysis (GSEA) was performed to explore the contribution of the biomarkers to PD progression. For this analysis, the default background gene set from the org.Hs.eg.db package (version 3.17.0) (40) was used as the reference. Additionally, Spearman correlation coefficients between each biomarker and all other genes in the GSE16134 dataset were calculated using the psych package (version 2.4.3) (https://CRAN.R-project.org/package=psych), and the genes were ranked by these correlation coefficients. GSEA was conducted using the clusterProfiler package (version 4.10.1), with thresholds including a normalized enrichment score (NES) greater than 1, an adjusted P-value below 0.25, and a P-value less than 0.05. The top five most significantly enriched pathways for each biomarker were visualized using the enrichplot package (version 1.22) (41). Furthermore, gene set variation analysis (GSVA) was performed to evaluate signaling networks in PD and control cohorts. The “C2: CP: KEGG gene sets” from the Molecular Signatures Database (MSigDB) (https://www.gsea-msigdb.org/) served as the reference gene collection. GSVA was executed using the GSVA package (version 1.50.0) (42) across all samples in the GSE16134 dataset. The limma package (version 3.58.1) was then applied to identify differentially regulated pathways between the PD and control groups (|t| > 2, P < 0.05).

### 2.7 Immune microenvironment analysis

The immune microenvironment of the PD and control groups was analyzed by determining the proportions of 22 immune cell subsets (43) in samples from the GSE16134 dataset, utilizing the CIBERSORT algorithm provided in the CIBERSORT package (version 0.1.0) (44). This algorithm was employed to calculate a probability, P, for the deconvolution of each sample using Monte Carlo sampling. Samples with P > 0.05 were excluded to ensure confidence in the results. A heatmap was then created using the corrplot package (version 0.92) (45) to display the distribution patterns of these 22 immune cell populations. Wilcoxon rank-sum testing was applied to assess statistical differences in immune cell infiltration between PD and control groups (P < 0.05). Additionally, Spearman correlation analysis, conducted using the psych package (version 2.4.3), examined the associations between biomarker candidates and differentially abundant immune cell populations, as well as the interrelationships among immune cell subsets across the entire GSE16134 dataset (|correlation values (r)| > 0.3, P < 0.05). A heatmap was generated using the corrplot package to visualize these correlations, and a separate heatmap using ggplot2 (version 3.5.1) was produced to show the associations between immune cell subsets and biomarkers.

### 2.8 Construction of regulatory networks

To explore the molecular regulatory networks influencing biomarker candidates, microRNA (miRNA)-mediated regulation was investigated using both the miRDB database (https://www.mirdb.org/) and the TargetScan database (http://www.targetscan.org/) to identify potential miRNA regulators targeting the biomarkers. Key miRNAs were identified by intersecting the predicted relationships from these two databases. Subsequently, upstream long non-coding RNAs (lncRNAs) for key miRNAs were predicted using the miRNet database (https://www.mirnet.ca/miRNet/). Transcription factors (TFs) that may target biomarkers were predicted using the NetworkAnalyst online platform (https://www.networkanalyst.ca/). The regulatory networks involving lncRNAs, key miRNAs, mRNAs, and TFs were visualized using Cytoscape software (version 3.10.2) (46).

### 2.9 Drug prediction and molecular docking

Candidate therapeutic compounds targeting the identified biomarkers were identified by querying the Drug Gene Interaction Database (DGIdb) (https://dgidb.genome.wustl.edu/). The interaction networks between these compounds and biomarkers were constructed and visualized using Cytoscape (version 3.10.2). Five compounds with the highest drug-target prediction scores and available three-dimensional structural information were selected for molecular docking simulations. These compounds were then searched in the PubChem database (https://pubchem.ncbi.nlm.nih.gov/) to obtain their three-dimensional conformations, while the protein structures of the biomarker targets were retrieved from the AlphaFold Protein Structure Database (https://alphafold.ebi.ac.uk/). Finally, molecular docking simulations between target proteins and selected compounds were performed using AutoDock Vina software (version 4.2.6) (47).

### 2.10 Processing of scRNA-seq data

The 10x scRNA-seq dataset from GSE171213 underwent quality control (QC) using the Seurat package (v5.1.0) (48), including quality control, normalization, dimensionality reduction, and batch effect correction. The PercentageFeatureSet function was used to compute key QC metrics: nFeature_RNA (the count of identified genes), nCount_RNA (total RNA transcript abundance per individual cell), and percent.mt (proportion of mitochondrial transcripts). The QC criteria were defined as follows: nFeature_RNA > 200, nCount_RNA > 3, and percent.mt < 40%, to remove low-quality cells or those with high mitochondrial content, thereby ensuring the reliability of downstream analyses. Subsequently, data normalization and batch integration were performed using the LogNormalize function combined with the “Harmony” algorithm. This process integrated the data from five PD and four healthy control samples into a unified expression matrix, along with the corresponding meta.data. Following data integration, normalization and dimensionality reduction were carried out according to the standard Seurat workflow. The expression matrix was normalized using the LogNormalize() method. The most variable 2,000 gene features were identified through the variance-stabilizing transformation (vst) method in the FindVariableFeatures function. Principal component analysis (PCA) was then performed on the 2,000 most variable genes *via* the RunPCA function, with scree plot inflection points helping to determine the optimal number of principal components (PCs) for further analyses. Subsequently, an unsupervised clustering analysis was conducted at a resolution of 0.5 using the FindClusters function to determine the number of cell clusters. Uniform Manifold Approximation and Projection (UMAP) was used to visualize the cell clusters in a two-dimensional map, providing an unbiased representation. The FindAllMarkers function was then applied to identify highly expressed genes for each cell cluster, using the parameters: |log_2_FC| > 1, |pct.1-pct.2| > 0.2, and adj.P < 0.05. The specific highly expressed genes for each cell cluster were compared with marker genes from the CellMarker database (http://117.50.127.228/CellMarker/) and from the literature (49), allowing for the annotation of cell clusters into distinct cell types.

### 2.11 Identification of key cell types

To assess differences in cell proportions between the PD and control groups for each annotated cell type, Wilcoxon tests were performed, identifying cell types with significant differences (P < 0.05) as differential cells. Functional enrichment analysis of differentially expressed cells was then conducted using the ReactomeGSA package (v 1.16.1) (50). Heatmaps illustrating the top 20 most significantly altered pathways among cell populations were generated using the corrplot package (v 0.92). Furthermore, t-SNE dimensionality reduction was performed using the RunTSNE function within Seurat (v5.1.0) to examine the expression patterns of biomarkers across individual annotated cell types. Bubble plots, created using the ggplot2 package (v 3.5.1), were employed to visualize biomarker expression profiles in distinct cell populations. The identification of key cell types was based on the following criteria: expression of at least two biomarkers, identification as differential cells in prior analyses, and significant roles in PD pathogenesis.

### 2.12 Cellular communication and pseudo-temporal trajectory analyses

The interaction dynamics among annotated cell types were analyzed using the aggregateNet function from the CellChat package (v1.6.1) (51), which quantified both the frequency and intensity of intercellular communication between primary cell populations and other defined subtypes. This analysis was followed by ligand-receptor interaction pair identification among the annotated cell types using the CellChat package (v1.6.1). Dimensionality reduction clustering was applied to analyze key cell types, following the established methodology for processing scRNA-seq data. The differentiation states of these key cell types were predicted, and their trajectories were mapped using the monocle package (v2.35.0) (52). Additionally, the DifferentialGeneTest function of monocle (v2.35.0) was utilized to examine the dynamic expression patterns of biomarkers during the differentiation of key cell types.

### 2.13 External validation of scRNA-seq data

To evaluate the robustness and generalizability of the identified biomarkers and key cell subsets, an independent periodontitis scRNA-seq dataset (GSE164241) was introduced for external validation. The data processing and analytical pipeline remained consistent with the primary analysis. Briefly, the Seurat package (v5.1.0) was used for quality control, normalization, selection of highly variable genes, principal component analysis (PCA), and UMAP dimensionality reduction and clustering. Following cell clustering, highly expressed genes for each cluster were identified, and cell type annotation was performed with reference to periodontitis scRNA-seq literature and the SingleR database. Subsequently, differences in the proportions of cell types between the periodontitis and healthy control groups were compared, and the expression distribution of the study-identified biomarkers across cell subsets in this dataset was visualized to validate the key findings.

### 2.14 Reverse transcription-quantitative real-time PCR

To further validate the expression levels of biomarkers between PD and control groups, RT-qPCR was performed. A total of 5 clinical samples from patients with PD and 5 samples from controls were collected at the First People’s Hospital of Yunnan Province (The gingival tissue samples from patients and healthy participants were mainly selected). Ethical approval for this investigation was granted by the Ethics Committee of the First People’s Hospital of Yunnan Province, with informed consent obtained from all participants. Total RNA was isolated from frozen tissue samples using the TRIzol kit (Vazyme Biotech Co., Ltd., Cat. R401-01, Nanjing, China) following standard protocols. RNA concentration was measured using a NanoPhotometer N50, and the results were used to determine the appropriate RNA amount for reverse transcription procedures. RNA templates underwent reverse transcription to complementary DNA (cDNA) using the Hifair^®^ III 1st Strand cDNA Synthesis SuperMix for qPCR Kit (Yeasen Biotechnology, Cat. 11141ES60, Shanghai, China) as per the manufacturer’s instructions. The resulting cDNA products were serially diluted 5–20 times using nuclease-free ddH2O. PCR amplification reactions were prepared with 3 µL of diluted cDNA, 5 µL of 2xUniversal Blue SYBR Green qPCR Master Mix (Saiweier Biotechnology Co., Ltd, Cat. G3326-05, Wuhan, China), and 1 µL of each forward and reverse primer (10 µM concentration). Biomarker primers were sourced from Sangon Biotech Co., Ltd., Shanghai, China, with sequence details provided in **Supplementary Table S2**. PCR amplification was performed for 40 cycles (pre-denaturation step excluded) using a CFX Connect real-time PCR system (BIO-RAD, XLFZ006), with thermal cycling conditions outlined in **Supplementary Table S3**. GAPDH served as the housekeeping gene reference, and relative gene expression levels were calculated using the 2^-△△CT^ comparative method. Bar graphs were constructed in GraphPad Prism software version 5 to visualize differential mRNA expression patterns of biomarkers comparing patients with PD and healthy controls.

### 2.15 Statistical analysis

Data analysis was performed in the R programming environment (version 4.3.1), using Wilcoxon rank-sum tests to assess inter-group statistical differences. Specifically, the limma statistical framework (version 3.58.1) was utilized to perform differential expression analysis. Functional enrichment analysis was conducted based on the clusterProfiler computational framework (version 4.10.1). Machine learning algorithms were implemented through the glmnet computational package (version 4.1.8), caret package (v 6.0.94), and Boruta package (v 8.0.0). A nomogram was constructed using the rms software package (v6.5.0). Correlation analysis was performed with the psych package (v 2.4.3). Quality control of single-cell RNA sequencing data was carried out using the Seurat package (v 5.1.0). Cell-cell communication analysis was conducted with the CellChat software package (v 1.6.1). Statistical differences between groups were determined using the Wilcoxon rank-sum test. For the quantitative PCR experiments, threshold cycle (Ct) values were analyzed using two-sided unpaired t-tests in GraphPad Prism 5. Statistical significance was defined at P < 0.05.

## 3 Results

### 3.1 Discernment of 5 candidate genes and exploration of their biological functions

**Figure 2:**
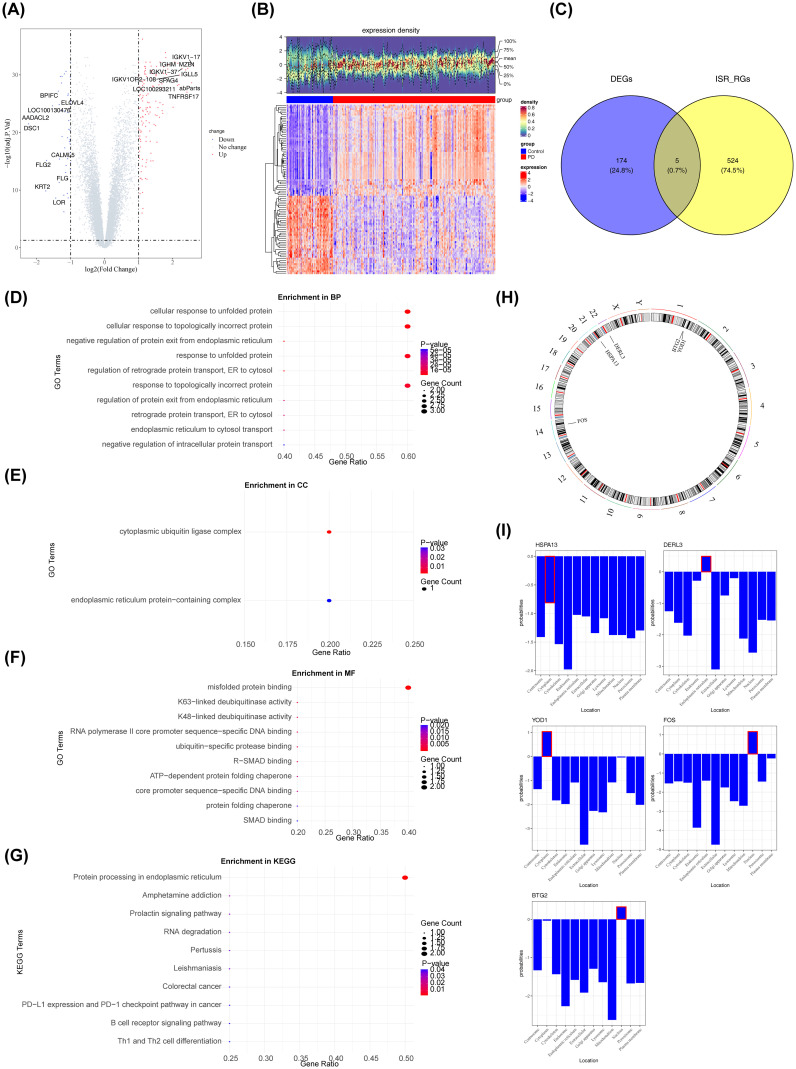
Analysis of differentially expressed genes (DEGs) in periodontitis patients. The screening criteria were FDR < 0.05 and |log2FC| > 1. **(A)** Volcano plot of the differential gene expression analysis. Red dots represented upregulated genes, blue dots represented downregulated genes, and gray dots represented genes with no significant difference or minimal fold change. The abscissa represented log_2_(Fold Change), which was the logarithm of the fold change with base 2. The Fold Change referred to the ratio of gene expression levels between the experimental group and the control group. The ordinate represented -log_10_(P value), which was the opposite number of the logarithm of the P value with base 10. The P value was used to measure the significance of differential expression, and the smaller the P value was, the stronger the statistical significance of the difference in gene expression between the two groups was. **(B)** The heatmap displayed the differentially expressed genes between periodontitis patients and the control group. The upper section presented the expression density heatmap, while the lower section showed the expression heatmap. Red indicated up-regulated genes, and blue indicated down-regulated genes. **(C)** Venn diagram illustrating the overlap between differentially expressed genes and genes related to the integrated stress response. **(D–F)** GO enrichment analysis of candidate genes. **(G)** Results of the KEGG enrichment analysis. **(H)** Chromosomal distribution of the candidate genes. **(I)** Subcellular localization analysis (All data sources and acquisition methods: Cell-PLoc 3.0 Online Prediction system).

A preliminary investigation of differential gene expression identified 179 DEGs between the PD and control groups, with 136 genes upregulated and 43 downregulated in the PD group (**Figures 2A, B**). Intersection analysis between these DEGs and 529 ISR-RGs revealed five candidate genes: BTG2, DERL3, FOS, HSPA13, and YOD1 (**Figure 2C**, **Supplementary Table S4**). Enrichment analyses were then performed to explore the signaling pathways associated with these genes. The results showed significant enrichment in 155 GO terms (P < 0.05), including 134 biological processes (BPs), 2 cellular components (CCs), and 19 molecular functions (MFs) (**Supplementary Table S5**). Notably, pathways significantly enriched (P < 0.05) included “cellular response to unfolded protein,” “cytoplasmic ubiquitin ligase complex,” and “misfolded protein binding” (**Figures 2D-F**). KEGG pathway analysis identified 18 significantly enriched pathways (P < 0.05), such as “protein processing in the endoplasmic reticulum (ER)” (**Figure 2G**, **Supplementary Table S6**). These results suggest the involvement of the candidate genes in critical cellular processes, including protein homeostasis, stress response, and ubiquitin-mediated degradation, implicating their role in ER function and cellular adaptation to proteotoxic stress. Structural analysis of BTG2, DERL3, FOS, HSPA13, and YOD1 revealed that BTG2 and YOD1 are located on chromosome 1, while DERL3, FOS, and HSPA13 are situated on chromosomes 22, 14, and 21, respectively (**Figure 2H**). Subcellular localization indicated that BTG2 and FOS are primarily nuclear, DERL3 is mainly located in the ER, and HSPA13 and YOD1 are predominantly cytoplasmic (**Figure 2I**).

### 3.2 Acquisition of 5 biomarkers: BTG2, DERL3, FOS, HSPA13, and YOD1

**Figure 3:**
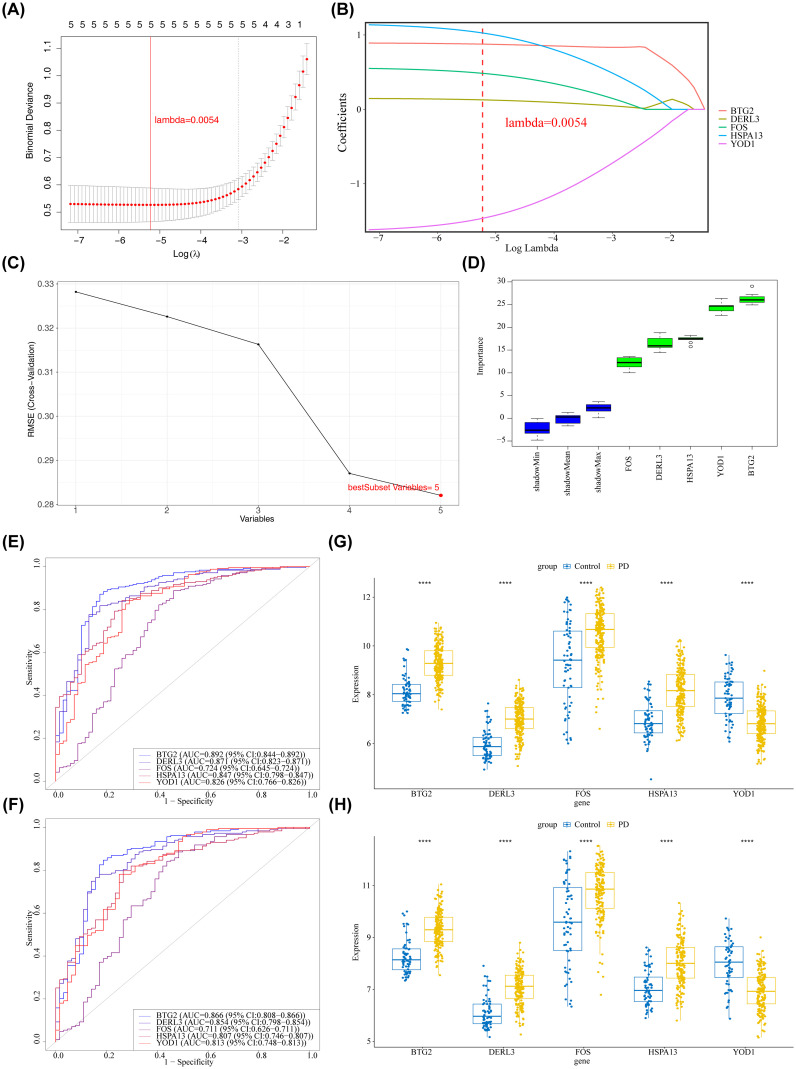
Identification of key and signature genes. **(A, B)** Results of feature gene selection using LASSO regression. In panel **(A)** the bottom x-axis represented log(lambda), the top x-axis indicated the number of non-zero coefficients, and the left y-axis represented the Binomial Deviance. The two dashed vertical lines corresponded to the minimum error and the most regularized model within one standard error of the minimum. Panel **(B)** showed the coefficient profiles of the variables across different log(lambda) values. **(C)** Results of feature gene selection using SVM-RFE. The x-axis indicated the number of selected features, and the y-axis represented the corresponding error rate. The red point indicated the number of features selected at the point of minimum error (or highest accuracy). **(D)** Feature importance ranking plot generated by the Boruta algorithm. The y-axis represented the feature importance score. Blue boxplots represented the minimum, average, and maximum Z scores of shadow attributes. Green boxplots represented confirmed important features. **(E, F)** ROC curve analysis of core genes in the training and validation sets. The x-axis represented the False Positive Rate (FPR); the y-axis represented the True Positive Rate (TPR). (**E**) Training set GSE16134. **(F)** Validation set GSE10334. **(G, H)** Expression levels of candidate biomarkers in PD and control samples from the training and validation sets. The x-axis represented genes, and the y-axis represented gene expression levels. ****p < 0.0001. **(G)** Training set GSE16134. **(H)** Validation set GSE10334.

The LASSO regression algorithm incorporated 5 candidate genes, yielding an optimal lambda value of 0.0054 that minimized the model error rate (**Figure 3A**). In this context, BTG2, DERL3, FOS, HSPA13, and YOD1 were identified with non-zero regression coefficients and designated as LASSO feature genes (**Figure 3B**). Furthermore, the SVM-RFE model achieved the lowest RMSE with 5 selected variables (**Figure 3C**), identifying the following as SVM-RFE feature genes: BTG2, DERL3, FOS, HSPA13, and YOD1. The Boruta algorithm identified 5 genes with importance surpassing ShadowMax, namely BTG2, DERL3, FOS, HSPA13, and YOD1, which were designated as Boruta feature genes (**Figure 3D**). Evidently, the aforementioned 3 machine learning algorithms identified consistent feature genes that aligned with the candidate genes (**Supplementary Figure S1**). Additionally, BTG2, DERL3, FOS, HSPA13, and YOD1 demonstrated their capacity to effectively differentiate PD samples from control samples, as evidenced by their AUC values exceeding 0.7 on the ROC curves in both GSE16134 and GSE10334 (**Figures 3E, F**). Remarkably, in the GSE16134 and GSE10334, the expression of BTG2, DERL3, FOS, and HSPA13 was notably elevated in the PD group compared to the control group (P < 0.001), while the expression of YOD1 was markedly reduced (P < 0.001) (**Figures 3G, H**). The results illustrated the stability and reliability of BTG2, DERL3, FOS, HSPA13, and YOD1, identifying them as biomarkers associated with PD.

### 3.3 Nomogram demonstrated favorable performance in assessing the diagnosis of PD

**Figure 4:**
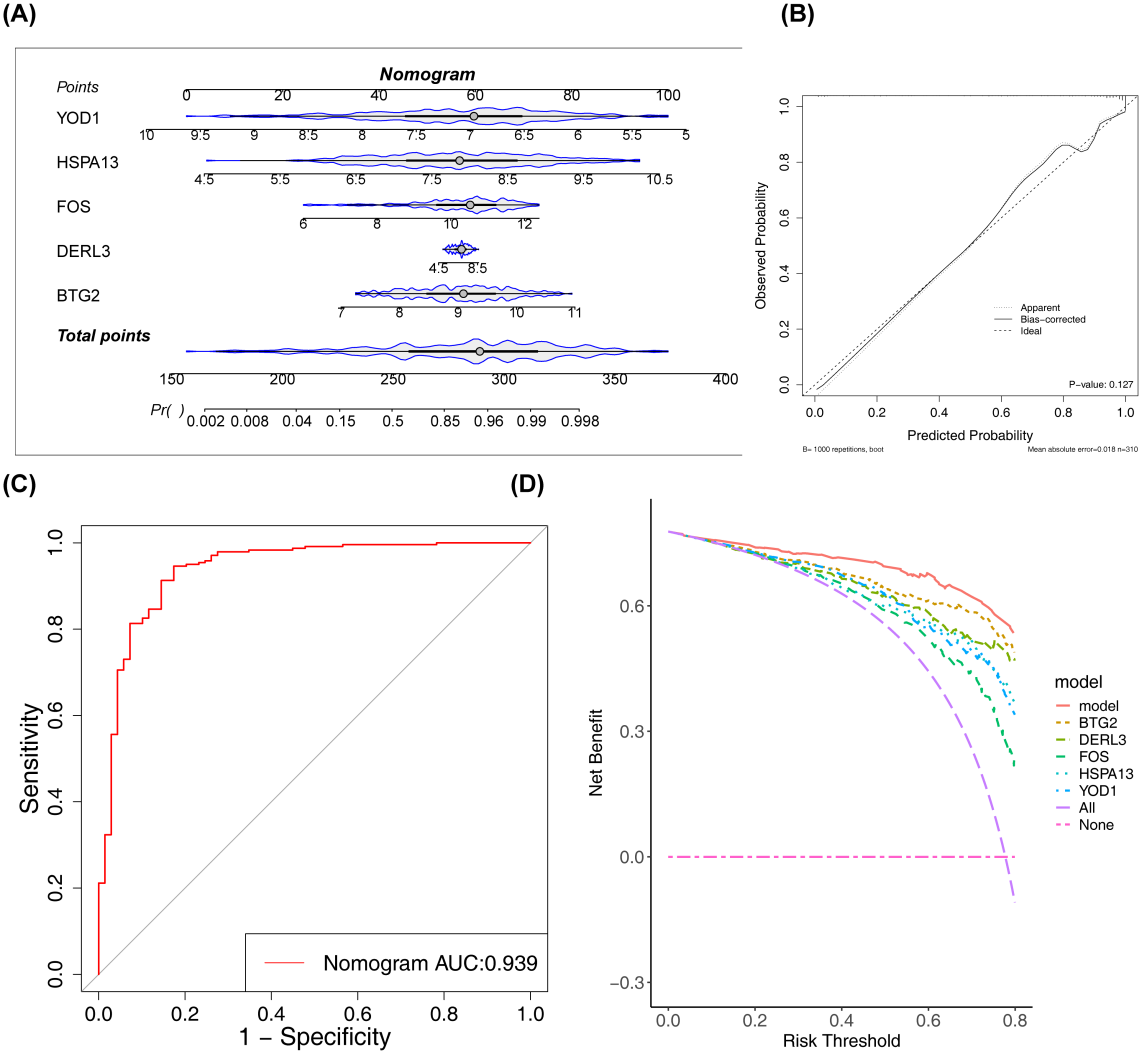
Construction and validation of the nomogram. **(A)** The constructed nomogram. **(B)** Calibration curve of the nomogram. The x-axis represented the predicted probability, and the y-axis represented the actual probability. The ‘Ideal’ line served as the reference. The ‘Apparent’ line represented the fit between the predicted and actual values before correction, and the ‘Bias-corrected’ line represented the fit after correction. **(C)** ROC curve of the nomogram. **(D)** Decision curve analysis (DCA) of the nomogram. The red line represented the nomogram model; the ‘None’ line (blue) represented the net benefit of intervening for no patients; the ‘All’ line (green) represented the net benefit of intervening for all patients.

A nomogram was developed to assess the diagnostic value of these five biomarkers in PD. The model demonstrated that higher total points for BTG2, DERL3, FOS, HSPA13, and YOD1 correlated with an increased likelihood of PD development (**Figure 4A**). The calibration curve (**Figure 4B**) supported the low diagnostic error rate of the nomogram (P = 0.127). Furthermore, the ROC curve indicated a high level of accuracy for the nomogram model, with an AUC value of 0.939 (**Figure 4C**). DCA revealed the highest net benefit for the nomogram (**Figure 4D**), suggesting its potential to enhance early PD diagnosis in clinical settings.

### 3.4 Functional analyses related to BTG2, DERL3, FOS, HSPA13, and YOD1

**Figure 5:**
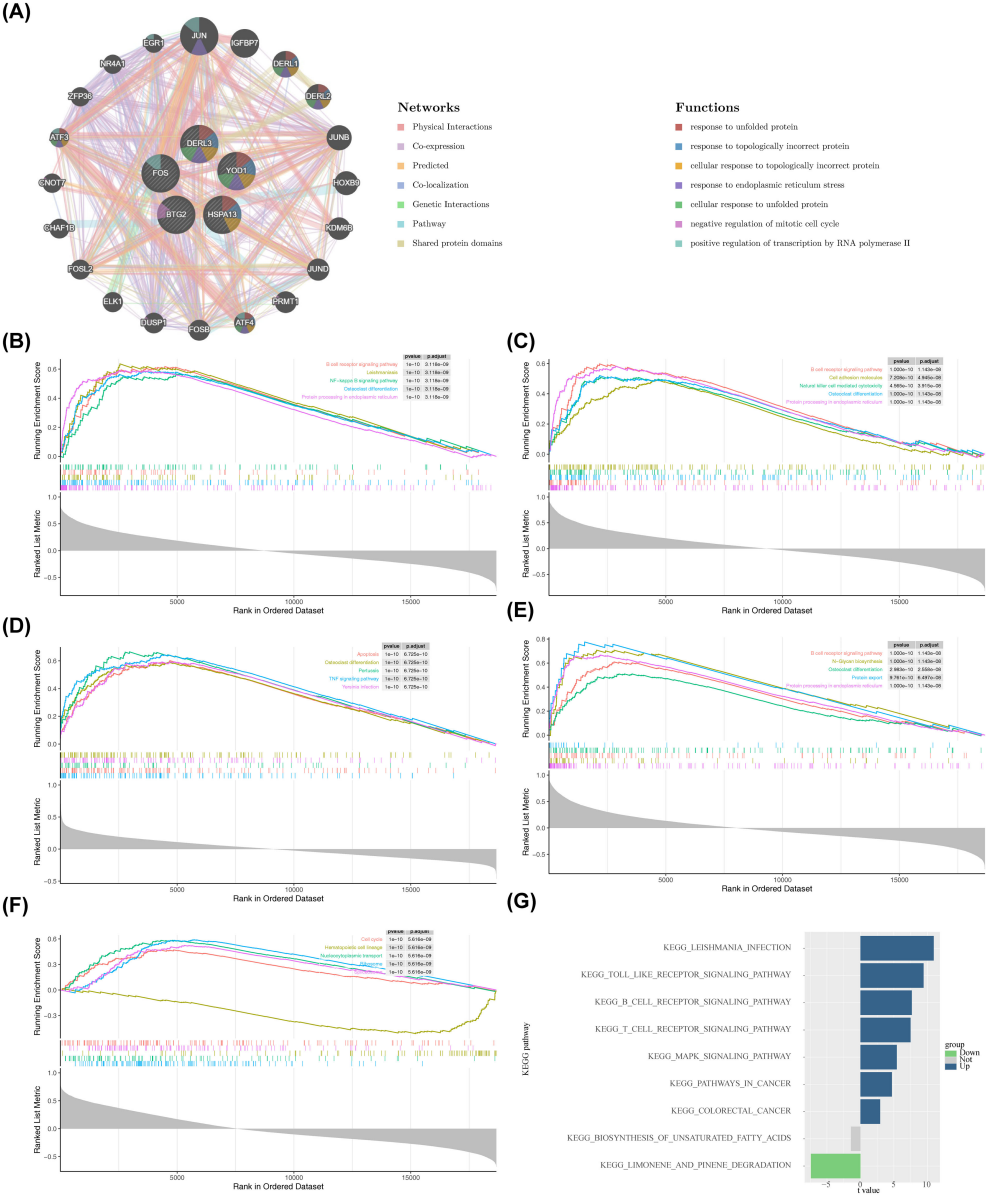
Enrichment analysis of biomarkers and construction of related networks. **(A)** GeneMANIA network construction results. **(B–F)** GSEA enrichment analysis of the biomarkers. The top plot in each panel showed the Enrichment Score (ES) profile, which reflected the degree to which a gene set was overrepresented at the top or bottom of a ranked list of genes. The middle section showed the barcode plot, indicating the positions of the gene set members in the ranked gene list. The bottom plot showed the distribution of the rank values (e.g., based on Signal-to-Noise ratio, S2N, shown in grey) for all genes in the ranked list, reflecting the magnitude of differential expression between the two groups. **(B)** BTG2. **(C)** DERL3. **(D)** FOS. **(E)** HSPA13. **(F)** YOD1. **(G)** GSVA enrichment results. The x-axis represented the t-value from limma differential analysis, and the y-axis represented the enriched pathways. Green indicated pathways significantly downregulated in the disease group, while blue indicated pathways significantly upregulated.

The functions associated with BTG2, DERL3, FOS, HSPA13, and YOD1 were further investigated. The GGI network included 25 genes, comprising BTG2, DERL3, FOS, HSPA13, and YOD1, as well as 20 other associated genes (**Figure 5A**). DERL3 and YOD1 shared numerous functional annotations with DERL1 and DERL2, including “response to unfolded protein” and “response to topologically incorrect protein.” Notably, BTG2, DERL3, FOS, and HSPA13 were co-enriched in the “osteoclast differentiation” pathway (**Figures 5B–E**). Additionally, BTG2, HSPA13, and DERL3 were jointly enriched in the “B cell receptor (BCR) signaling pathway” and “protein processing in the ER.” These results suggest that BTG2, DERL3, and HSPA13 may be involved in essential biological processes, such as osteoblast differentiation, immunomodulation, and protein processing, thereby influencing the initiation and progression of PD. Moreover, YOD1’s primary involvement in pathways related to the “cell cycle,” “nucleocytoplasmic transport,” and “ribosome” suggests its critical role in PD pathogenesis by regulating cell proliferation, molecular transport, and protein synthesis (**Figure 5F**). Furthermore, GSVA was performed to explore pathway enrichment differences between the PD and control groups. The PD group exhibited activation of 7 pathways, including the “Toll-like receptor signaling pathway” (t > 2), while pathways such as “biosynthesis of unsaturated fatty acids” and “limonene and pinene degradation” were inhibited (t < 2) (**Figure 5G**). These results indicate that the activation of immune-related pathways, such as the Toll-like receptor signaling pathway, reflects enhanced innate immune responses and exacerbated inflammation in PD, highlighting the significant role of immune reactions in the disease.

### 3.5 Immune cells distribution and correlation analysis of BTG2, DERL3, FOS, HSPA13, and YOD1

**Figure 6:**
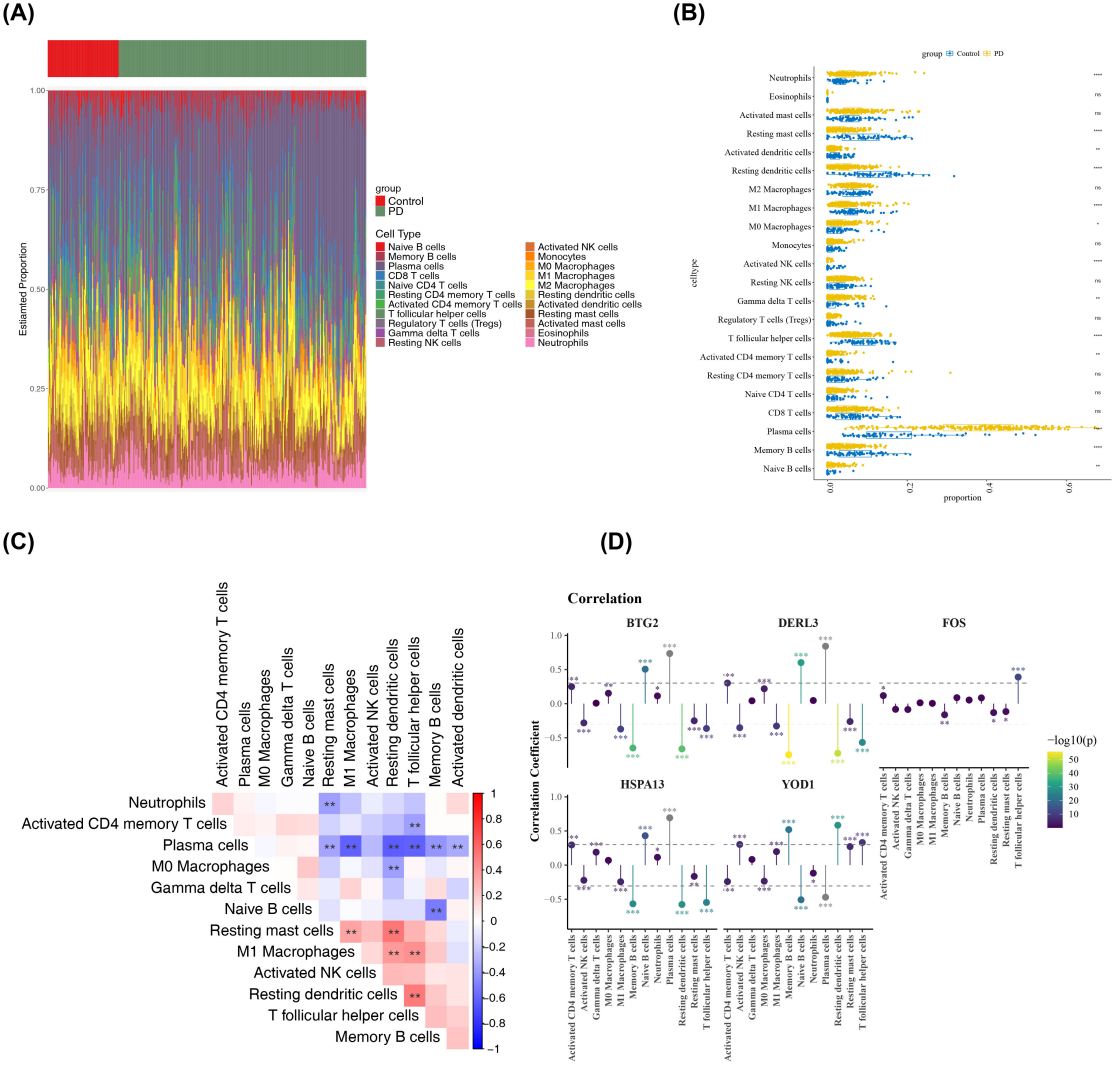
Analysis of the immune microenvironment. **(A)** Infiltration abundance of 22 immune cell types in all PD and control samples. The x-axis represented samples, and the y-axis represented the proportion of immune cells. The top color bar indicated the sample type, with green for PD samples and red for control samples. **(B)** Differences in the infiltration of each immune cell type between PD and control samples. The x-axis represented the GSEA score, and the y-axis represented the immune cell types. Yellow represented PD samples, and blue represented control samples. **p < 0.01, ****p < 0.0001, ns: not significant. **(C)** Correlations among the differentially infiltrated immune cells in PD and control samples. Red indicated positive correlation, blue indicated negative correlation. **p < 0.01, **(D)** Correlations between biomarkers and differentially infiltrated immune cells. Yellow indicated positive correlation, Purple indicated negative correlation, the vertical axis represented the correlation coefficient score. *p < 0.05, **p < 0.01, ***p < 0.001.

An analysis of the immune microenvironment between the PD and control groups was then conducted. A heatmap illustrating the infiltration levels of 22 distinct immune cell types is presented in **Figure 6A**. Significant differences (P < 0.05) in immune cell infiltration were detected between the PD and control groups across 13 immune cell subtypes, including resting mast cells (**Figure 6B**). The most notable positive correlation was observed between resting mast cells and resting dendritic cells (cor = 0.52, P < 0.01), while the strongest negative correlation was found between plasma cells and M1 macrophages (cor = -0.60, P < 0.01) (**Figure 6C**, **Supplementary Table S7**). Additionally, DERL3 showed the most significant positive association with plasma cells (cor = 0.84, P < 0.001) and the most substantial inverse relationship with resting dendritic cells (cor = -0.78, P < 0.001) (**Figure 6D**, **Supplementary Table S8**). Interestingly, YOD1 exhibited contrasting correlation patterns compared to BTG2, DERL3, and HSPA13 when assessed against the same immune cell populations, such as plasma cells, T follicular helper cells, and M1 macrophages.

### 3.6 Molecular regulatory networks and drug interactions involving BTG2, FOS, HSPA13, and YOD1 in PD treatment

**Figure 7:**
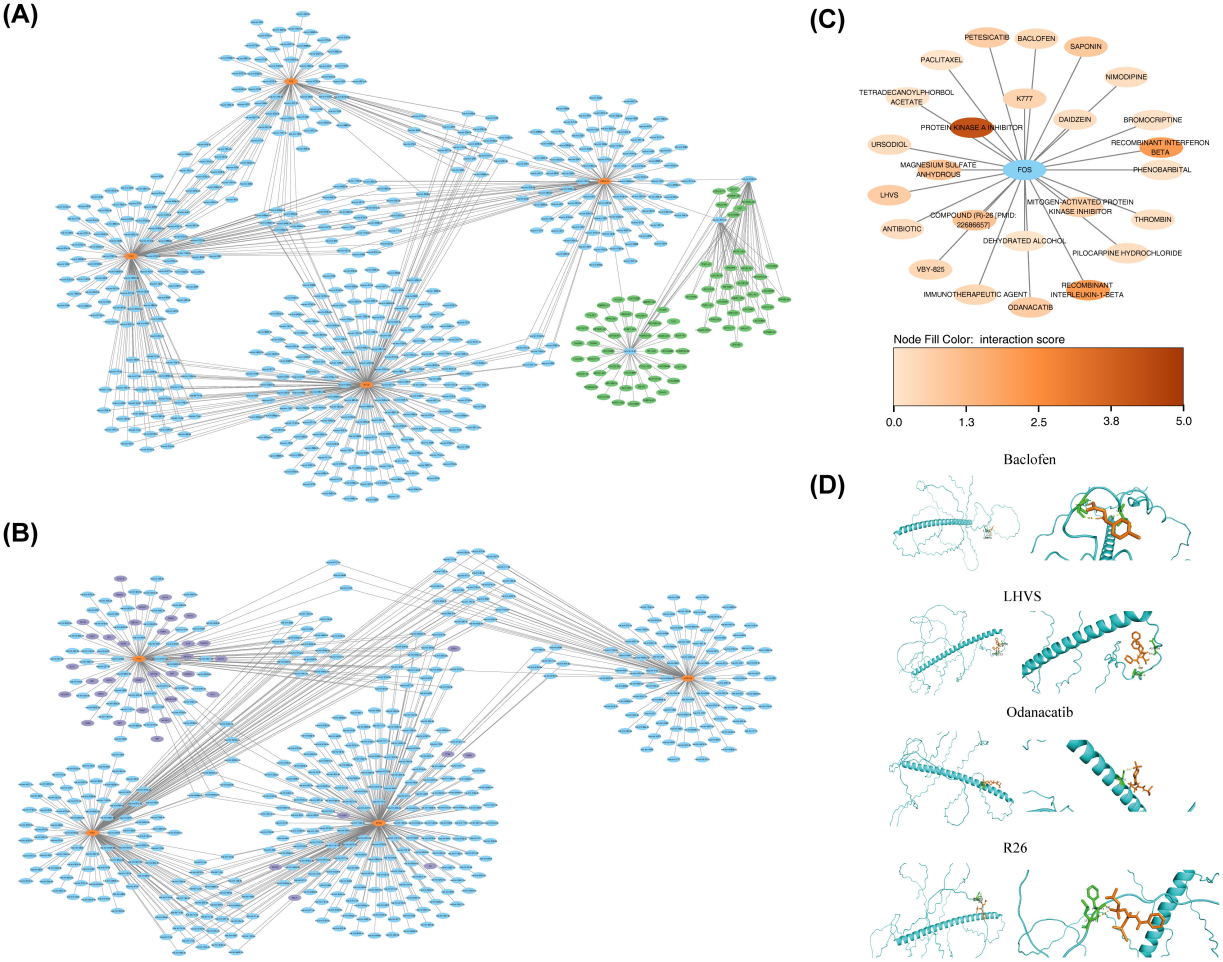
Construction of biomarker regulatory networks and drug prediction. **(A)** lncRNA-miRNA-biomarker regulatory network. Orange-red nodes represented biomarkers, blue nodes represented miRNAs, and green nodes represented lncRNAs. **(B)** TFs-biomarkers-miRNA regulatory network. Orange-red nodes represented biomarkers, blue nodes represented miRNAs, and purple nodes represented TFs (Transcription Factors). **(C)** Drug-target interaction network. Blue nodes represented target proteins, and other nodes represented drugs. **(D)** Molecular docking results for predicted drugs and proteins.

Molecular regulatory networks provided additional insights into the regulatory factors influencing BTG2, DERL3, FOS, HSPA13, and YOD1. A lncRNA-key miRNA-mRNA network was constructed, encompassing four biomarkers (BTG2, FOS, HSPA13, and YOD1), 498 key miRNAs, and 78 lncRNAs (**Figure 7A**). For example, HCG18 regulated HSPA13 expression through modulation of hsa-miR-9-5p. Additionally, BTG2 and FOS were predicted to be regulated by 39 TFs, with ESR1 identified as a common TF in both predictions (**Figure 7B**).Then, All five key ISR biomarkers (FOS, BTG2, DERL3, HSPA13, YOD1) were submitted to the DGIdb database for screening. FOS was identified as a “druggable” gene with known targeting relationships; therefore, subsequent drug analysis primarily focused on FOS and its matched compounds. In the DGIdb database, FOS was associated with 25 related drugs (**Figure 7C**). Among these, the five drugs with the highest interaction scores with FOS and known three-dimensional structures were compound (R)-26 (R26), Odanacatib, Petesicatib, LHVS, and Baclofen (**Supplementary Table S9**). The 3D structure of the FOS protein, designated as AF-P01100-F1-v4, was obtained from AlphaFold. Molecular docking simulations were then performed between the FOS protein and these five drugs. While the docking of FOS with petesicatib was unsuccessful, the binding energies for FOS with R26, odanacatib, LHVS, and baclofen were -4.4 kcal/mol, -5.6 kcal/mol, -5.2 kcal/mol, and -4.2 kcal/mol, respectively (**Supplementary Table S10**). These molecular docking results are illustrated in **Figure 7D**, showing the formation of hydrogen bonds in all docking processes. The binding free energies of FOS with both odanacatib and LHVS were below -5 kcal/mol, suggesting strong affinity between the TLR2 receptor and the ligands of these two drugs. These results indicate that odanacatib and LHVS may have potential applications in PD treatment.

### 3.7 T cells were identified as the key cell type

**Figure 8:**
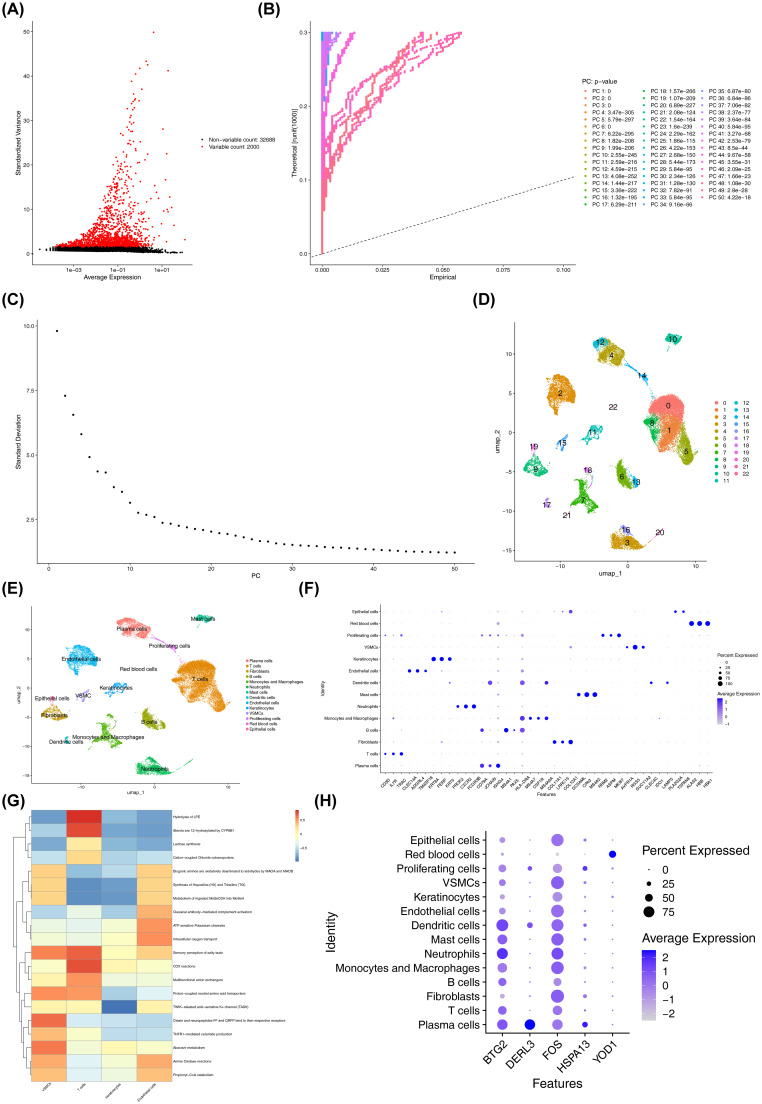
Quality control, dimensionality reduction, and clustering analysis of single-cell data. **(A)** Distribution of highly variable genes. Each point represented a gene, the x-axis represented the mean expression, and the y-axis represented the standard deviation. **(B)** PCA results highlighting significant principal components. The significance of each PC was assessed by comparing the distribution of p-values for all genes on that PC against a uniform distribution. The p-value distribution (solid line) was strongly skewed to the left compared to the uniform distribution (dashed line), indicating significant biological signal in that PC. **(C)** PCA scree plot. The x-axis represented the PCA dimension number, and the y-axis represented the standard deviation. **(D)** Visualization of cell cluster classification. Points (cells) were colored by their assigned cluster in the UMAP plot, where closer points indicated greater similarity. **(E)** Cell types after annotation. **(F)** Expression levels of the annotated marker genes across cell clusters. The x-axis represented the marker genes used for cell type annotation, and the y-axis represented the cell types. **(G)** Functional enrichment of key cell clusters. The x-axis represented cell types, the y-axis represented enriched pathways, and the color indicated the enrichment score. Redder colors indicated higher scores. **(H)** Expression of biomarkers in different cell types. The x-axis represented the biomarkers, and the y-axis represented the different cell types.

Before QC, the GSE171213 dataset contained 45,191 cells and 34,688 genes. After QC, the number of cells was reduced to 34,683, while the gene count remained stable at 34,688 (**Supplementary Figure S2A**). Following standard data processing, 2,000 highly variable genes were identified (**Figure 8A**). PCA revealed clear segregation between the PD and control groups within GSE171213, suggesting substantial differences between the two cohorts (**Supplementary Figure S2B**). The top 30 PCs (P < 0.05) were selected for downstream analysis (**Figures 8B, C**). UMAP clustering identified 23 distinct cellular clusters (**Figure 8D**). Marker gene annotation categorized 14 different cell populations, including plasma cells, T cells, fibroblasts, and others (**Figure 8E**). The specific marker genes associated with each annotated cell type are detailed in **Supplementary Table S11** and **Figure 8F**. Significant differences in the proportions of VSMCs, T cells, keratinocytes, and endothelial cells were observed between the PD and control groups (P < 0.05) (**Supplementary Figure S2C**). These cell types were identified as differential cells. Furthermore, substantial differences in pathway enrichment for VSMCs, T cells, keratinocytes, and endothelial cells were observed (**Figure 8G**). For instance, “hydrolysis of LPE” and “12-hydroxylation of sterols by CYP8B1” were significantly enriched in T cells, with lower enrichment levels in other cell types. As a result, T cells were identified as a key cell type due to their expression of at least two biomarkers, particularly BTG2 and FOS (**Figure 8H**, **Supplementary Figure S2D**), and their documented involvement in PD mechanisms as described in the literature (53).

### 3.8 Cellular communication and pseudo-temporal trajectory analyses of T cells

**Figure 9:**
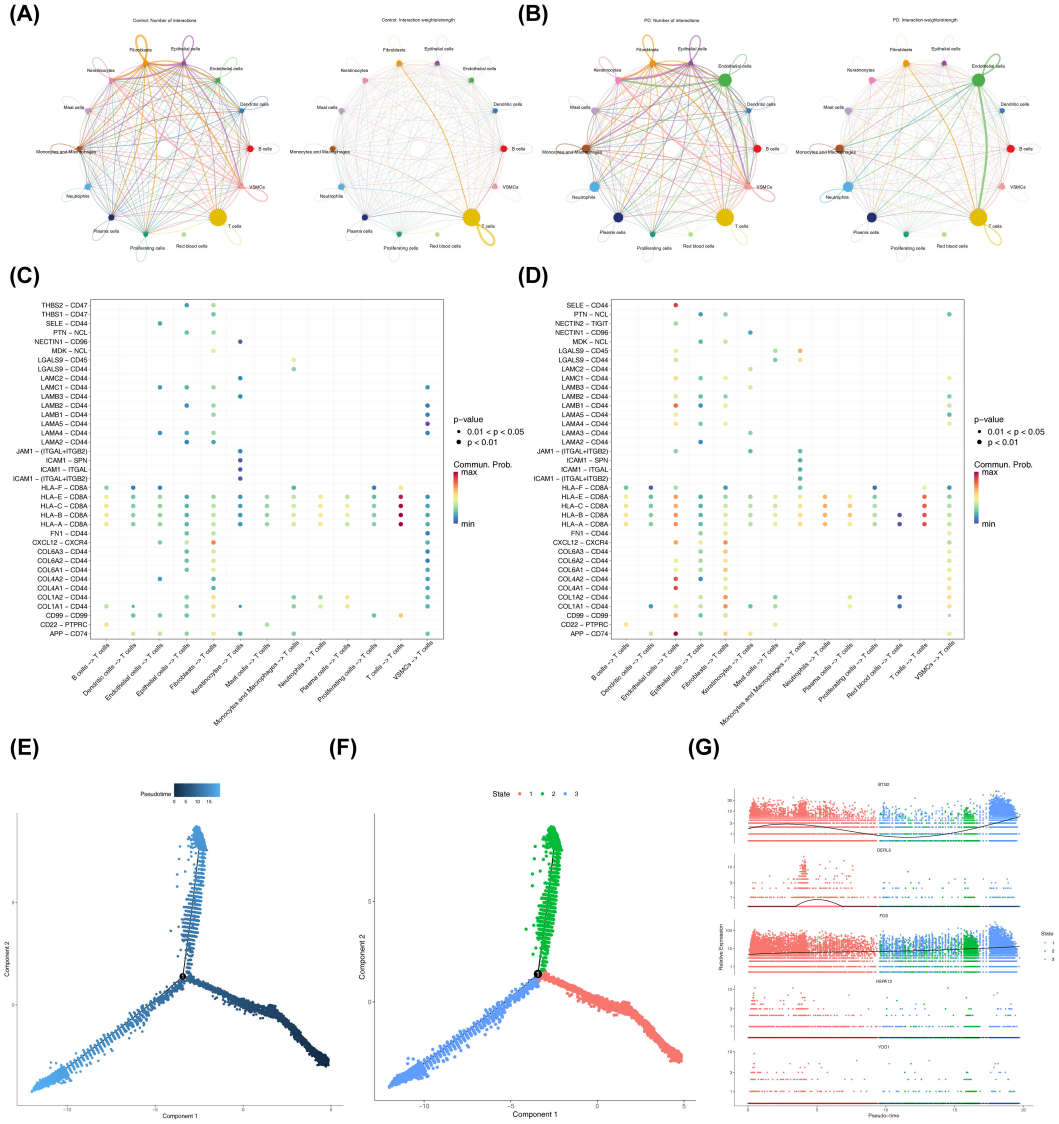
Cell-cell communication and pseudotime analysis. **(A, B)** Network diagrams showing the number and strength of differential cellular interactions between PD and control samples. The left side of the plot showed the number of interactions between cell types, and the right side showed the computed interaction strength. The thickness of the connecting lines represented the quantity or strength. **(A)** Cell-cell communication in control samples. **(B)** Cell-cell communication in disease samples. **(C, D)** Ligand-receptor interactions between different cell types in PD and control samples. The x-axis represented the interacting cell pairs and direction, and the y-axis represented the ligand-receptor pairs. The color of the bubbles represented the interaction probability, and the size represented the significance. **(C)** Control samples. **(D)** Disease samples. **(E)** Pseudotime trajectory analysis of T cells. **(F)** Pseudotime trajectory plot of T cells. **(G)** Expression trends of biomarkers in key cell clusters across different pseudotime stages. Colors represented different samples, the x-axis numbers represented inferred time points along the trajectory, and the y-axis represented gene expression levels.

Analysis of cellular communication revealed complex interrelationships among the 14 annotated cell types. Compared to the control cohort, communication intensity between T cells and endothelial cells was notably stronger in the PD group (**Figures 9A, B**). In the control cohort, the interaction pairs HLA-E-CD8A, HLA-C-CD8A, and related HLA-CD8A combinations were most prevalent in T cell-T cell communication pathways (**Figure 9C**, **Supplementary Figure S3A**). In contrast, in the PD group, the interaction pairs involving HLA-E-CD8A, HLA-C-CD8A, HLA-B-CD8A, and HLA-A-CD8A showed higher probability in the T cell autocrine signaling pathway (**Figures 9D**, **S3B**). Notably, in the PD group, the APP-CD74 interaction pair displayed the highest probability in the pathways from endothelial cells to T cells. Overall, the relative strength of signaling in endothelial cells was greater in the PD group compared to controls (**Supplementary Figure S3C**). Next, a detailed pseudo-temporal trajectory analysis of T cells was performed. The differentiation trajectory of T cells, visualized in **Figure 9E**, demonstrated a temporal progression from right to left, with the darkest blue representing the earliest stages of differentiation. As shown in **Figures 9F**, T cells were classified into three distinct states across different time points. The expression of BTG2 increased gradually in the early stages of T cell differentiation, decreased, and then rose again in the mid-to-late stages (**Figure 9G**). DERL3 expression remained stable at first, then slowly increased before decreasing back to baseline, where it stayed constant. FOS expression steadily increased throughout the differentiation process, while HSPA13 and YOD1 did not show fluctuations during T cell differentiation.

### 3.9 External validation results of the scRNA-seq analysis

Using the independent dataset GSE164241, the main research findings were successfully validated externally. After quality control, a total of 95,162 high-quality cells and 23,816 genes were obtained for subsequent analysis (**Supplementary Figures S4A, B**). Following standardization, dimensionality reduction (**Supplementary Figures S4C–E**), and clustering, all cells were annotated into 13 major cell types, including T/NK cells, myeloid cells, B cells, plasma cells, keratinocytes, among others (**Supplementary Figure S5A**). The expression patterns of cell type marker genes are shown in **Supplementary Figure S5B**.

Comparison of cell type proportions between the periodontitis and healthy control groups revealed that plasma cells were significantly enriched in the periodontitis group (**Supplementary Figures S5C–E**). This trend aligned with the changes in the immune landscape observed in the primary GSE171213 dataset. Further observation of biomarker expression showed high expression of BTG2 and FOS in key immune populations such as T cells (**Supplementary Figure S5F**). Considering their known roles in periodontitis and their identification as differential cells in this study, T cells were reaffirmed as the key cell type.

In summary, external validation based on the independent dataset GSE164241 showed high consistency with the results from the primary GSE171213 dataset regarding changes in cellular composition, enrichment trends of key immune cells, and the expression patterns of ISR-related biomarkers. This validation significantly strengthened the robustness and generalizability of the conclusion that ISR-related markers and T cells play a central role in the periodontitis immune microenvironment.

### 3.10 RT-qPCR analysis of BTG2, DERL3, FOS, HSPA13, and YOD1

**Figure 10:**
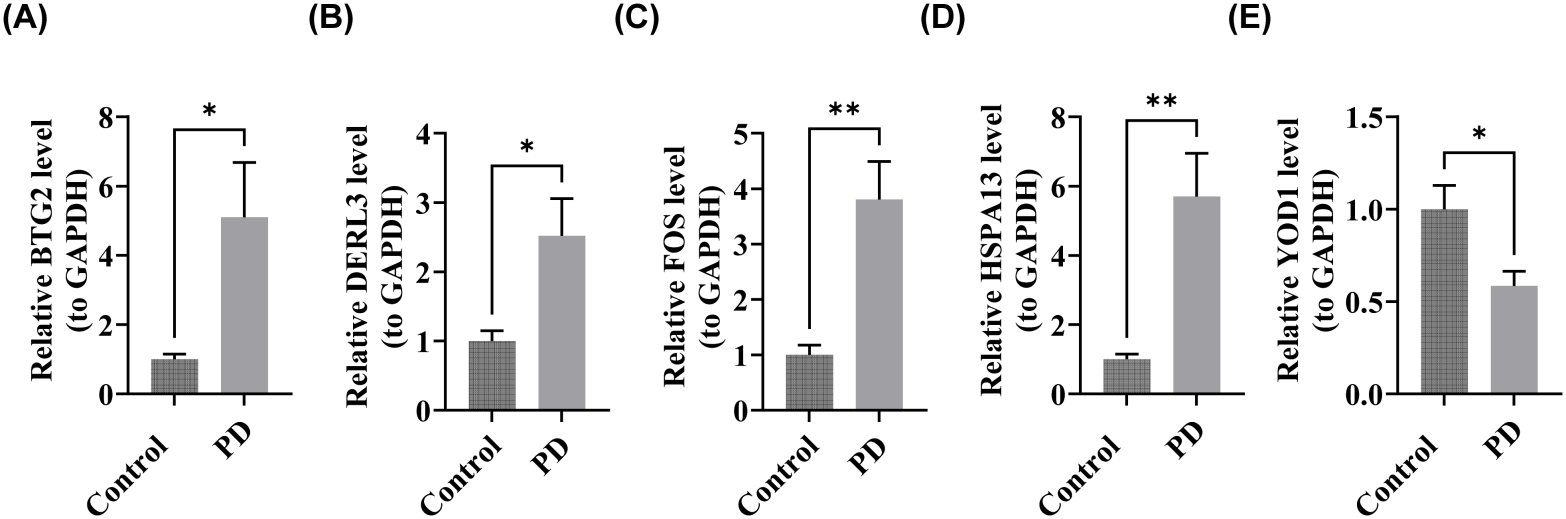
Validation of biomarker expression. **(A)** BTG2, **(B)** DERL3, **(C)** FOS, **(D)** HSPA13, **(E)** YOD1. “*”<0.05, “**”<0.01.

After total RNA extraction, concentration assessments confirmed that all samples met acceptable RNA concentration standards (**Supplementary Table S12**). Quantitative RT-PCR analysis revealed significant upregulation of BTG2, DERL3, FOS, and HSPA13 in patients with PD compared to healthy controls (P < 0.05) (**Figures 10A–D**). In contrast, YOD1 was significantly downregulated in the PD cohort (P < 0.05) (**Figure 9E**). These expression profiles were consistent with computational predictions from the GSE16134 and GSE10334 datasets for these five biomarkers, thus validating both the bioinformatics approach and experimental findings.

## 4 Discussion

PD is a plaque-induced inflammatory disease driven by oral microbiota dysbiosis and immune dysregulation (54). The ISR, which maintains proteostasis through eIF2α phosphorylation-mediated translational control, plays a critical role in cellular fate decisions, balancing survival and apoptosis (55). Given its role in regulating the cell survival-death equilibrium, ISR has emerged as a promising therapeutic target. Although its involvement in PD has not been explored, modulating the ISR could provide novel insights into PD pathology and therapeutic strategies. This study systematically constructed an ISR gene expression profile in periodontitis, spanning from tissue-level to single-cell resolution, through integrated multi-omics analysis. We not only identified a novel set of ISR-related biomarkers—BTG2, DERL3, FOS, HSPA13, and YOD1—but also revealed their potential associations with periodontitis-specific pathological features, such as osteoclast differentiation and the RANKL/OPG signaling pathway. At the single-cell level, this research for the first time pinpointed T cells as targets of ISR gene regulation and uncovered dynamic patterns of ISR signaling during immune cell differentiation. These findings extend beyond the conventional understanding of the integrated stress response in inflammatory processes, providing new insights into the molecular mechanisms of periodontitis and establishing a theoretical foundation for developing therapeutic strategies targeting the ISR pathway.

BTG2 (B-cell translocation gene 2), a tumor suppressor gene in the BTG/TOB family, regulates cell proliferation, differentiation, and apoptosis (56, 57). Emerging evidence identifies BTG2 as a molecular link between diabetic kidney disease (DKD) and PD, where it modulates autophagy via mTORC1 pathway inhibition, potentially suppressing epithelial-mesenchymal transition (EMT) (58).It is noteworthy that the mTORC1 pathway not only regulates autophagy but also modulates the activation of the NF-κB signaling pathway (59), which itself plays a critical regulatory role in both inflammatory responses and osteoclast differentiation in periodontitis (60). In the present study, the significant upregulation of BTG2 in periodontitis patients suggests its potential involvement in the pathological process as a stress-responsive molecule. Based on previous research, we hypothesize that altered BTG2 expression may indirectly influence the inflammatory state and osteoclastogenesis in periodontitis through modulation of the mTORC1 pathway.

DERL3 (Derlin-3), an ER-resident protein, mediates the ER-associated degradation (ERAD) of misfolded proteins during ER stress (61). Recent studies have confirmed that DERL3 participates in the pathogenesis of periodontitis by modulating the TLR4/MyD88 pathway through KAT3B-regulated succinylation modification (62). The TLR4/MyD88 pathway is a well-established regulatory axis in periodontitis (63). In our study, the significant upregulation of DERL3 in periodontitis patients suggests its potential role as a molecular link connecting endoplasmic reticulum stress to periodontal inflammatory responses. By maintaining endoplasmic reticulum homeostasis, DERL3 may exert regulatory effects on key inflammatory signaling pathways such as TLR4/MyD88. This finding provides a new research direction for exploring the molecular mechanisms of periodontitis from the perspective of proteostatic regulation.

The FOS protein (FBJ murine osteosarcoma viral oncogene homolog) is a core component of the TF AP-1 complex. It typically forms heterodimers with Jun proteins to bind specific DNA sequences and regulate gene expression. The FOS gene is essential in regulating cell proliferation, differentiation, apoptosis, and stress responses, especially in response to growth factors, cytokines, and external stimuli such as UV radiation and oxidative stress. Additionally, it contributes to neural plasticity, immune regulation, and tumorigenesis, with dysregulation linked to various cancers and diseases (64). In the complex pathological milieu of periodontitis, FOS is likely implicated in the initiation and progression of the disease through multiple mechanisms. First, as an effector molecule in the RANKL signaling pathway (65), the heterodimer formed by FOS and Jun proteins can respond to RANKL-activated MAPK pathways (p38, ERK1/2, and JNK) and cooperate with NFATc1 to regulate the expression of osteoclast-specific genes (66). More importantly, dysregulation of FOS may be linked to sustained bone destruction in periodontitis. For instance, downregulation of LRP5 has been shown to exacerbate periodontal inflammation and bone loss by impairing PI3K/c-FOS signaling, further consolidating the role of FOS within this signaling axis (67). In summary, FOS likely contributes to the pathogenesis of periodontitis by modulating osteoclast differentiation and associated signaling pathways, providing a new direction for further exploration of the molecular mechanisms underlying periodontal bone destruction.

HSPA13 (Heat Shock Protein Family A Member 13), a member of the Hsp70 family, is crucial for protein folding, transport, and degradation to maintain cellular proteostasis. It is upregulated in response to stressors such as heat shock and oxidative stress, binding to unfolded or misfolded proteins to prevent aggregation and facilitate refolding or degradation. HSPA13 is also involved in apoptosis, immune regulation, and signal transduction, with emerging roles in cancer and neurodegenerative diseases (68–70). While studies linking HSPA13 to PD are limited, this study reveals for the first time a significant upregulation of HSPA13 in periodontitis tissues, suggesting its potential involvement in disease pathogenesis. Converging with existing evidence that HSPA13 promotes NF-κB-mediated transcription (70), we hypothesize that it may contribute to the regulation of the local inflammatory response by modulating key signaling pathways such as NF-κB. These findings provide novel insights into integrated stress response biomarkers in periodontitis and propose a new avenue for investigating their functional mechanisms.

YOD1 (Yeast OTU1 Deubiquitinating Enzyme 1 Homolog) encodes a deubiquitinating enzyme and belongs to the OTU (ovarian tumor-associated protease) family. YOD1 regulates protein stability, function, and degradation by removing ubiquitin chains from target proteins, participating in various cellular processes such as cell cycle regulation, stress response, and signaling (71). Bioinformatics analyses predict that YOD1 may become an important target for the diagnosis and treatment of PD (72, 73).YOD1, known for its key function in maintaining tissue homeostasis and repair during inflammation (74), is thus implicated in sustaining periodontal tissue equilibrium, offering a new clue to its potential role in periodontitis.

The detection of clinical biomarkers for periodontitis typically relies on gingival crevicular fluid (GCF), saliva, or blood samples (75). GCF, derived directly from the periodontal lesion microenvironment, represents a rich source of protein and RNA biomarkers for periodontal diagnosis (76). Saliva offers the advantage of non-invasive collection, making it more suitable for large-scale screening (77), while blood samples can reveal systemic disease associations and are among the most frequently analyzed bio-specimens (78). In this study, BTG2, DERL3, FOS, and HSPA13 were significantly upregulated, and YOD1 was significantly downregulated in the gingival tissues of periodontitis patients, confirming their detectable presence locally. Consequently, future research will aim to detect these biomarkers in GCF, saliva, and blood to validate their expression levels in biofluids and thereby facilitate clinical translation.

The biomarker function analysis (section 3.4) revealed that BTG2, DERL3, FOS, and HSPA13 are co-enriched in the “osteoclast differentiation” pathway, while BTG2, DERL3, and HSPA13 are also co-enriched in the “BCR signaling pathway” and “protein processing in the ER.” Osteoclast differentiation is a critical process in bone metabolism, involving the transformation of mononuclear precursor cells into mature, multinucleated osteoclasts capable of bone resorption. This differentiation process is primarily controlled by two key regulators: Receptor Activator of NF-κB Ligand (RANKL) and Macrophage Colony-Stimulating Factor (M-CSF). These molecules activate critical TFs, including NFATc1 and c-FOS, which regulate osteoclast-specific gene expression (79). In PD—an inflammatory condition targeting tooth-supporting tissues—osteoclast differentiation plays a significant role in alveolar bone loss (80). The inflammatory environment in PD, marked by elevated levels of TNF-α, IL-1β, and IL-6, enhances RANKL expression and osteoclast differentiation (81). This leads to increased bone resorption and subsequent tooth loss. Periodontal pathogens can also directly stimulate osteoclast differentiation through mechanisms such as Toll-like receptor activation (82). Understanding the mechanisms of osteoclast differentiation in PD is essential for designing targeted interventions to mitigate alveolar bone loss and improve periodontal health. The BCR signaling pathway regulates B cell activation, differentiation, and survival. Antigen binding initiates a phosphorylation cascade involving protein tyrosine kinases, phospholipase C, and adaptor proteins (83), modulating gene expression, cytoskeletal dynamics, and metabolic reprogramming to coordinate immune responses (84, 85). Concurrently, ER protein homeostasis is maintained through a coordinated system of chaperones, processing enzymes, and QC mechanisms. This system facilitates proper protein folding, post-translational modifications, and targets misfolded proteins for ERAD (86). The co-enrichment of BTG2, DERL3, and HSPA13 in these pathways implicates their functional interplay in regulating periodontal immune responses and stress resilience.

Results from section 3.5 identified 13 differentially expressed immune cell types between the PD and control groups. Among these, YOD1 exhibited the strongest positive correlation with plasma cells (r = 0.84, P < 0.001) and the strongest negative correlation with resting dendritic cells (r = -0.78, P < 0.001). The periodontal immune microenvironment consists of diverse cell populations, extracellular matrix components, and cytokines that interact within a complex regulatory network (87). Plasma cells, terminally differentiated B lymphocytes, are central to adaptive immunity through large-scale antibody production, neutralizing pathogens and facilitating their clearance (88). These cells are localized in bone marrow, lymphoid tissues, and inflammatory sites (89, 90). In PD, plasma cells contribute to inflammation *via* pathogen-specific antibodies, complement activation, and pro-inflammatory cytokine secretion, driving chronic disease progression (91).

Resting dendritic cells (immature DCs) play a pivotal role in antigen processing and immune response regulation. In PD, bacterial components activate these cells to capture antigens, migrate to lymph nodes, and prime T-cell-mediated adaptive immunity (92, 93).

In the context of drug prediction and molecular docking analysis, this study focused on the interactions between the FOS protein and various small-molecule compounds. The results revealed that both odanacatib and LHVS exhibit high binding affinity with FOS (binding energies < -5.0 kcal/mol), suggesting their potential to modulate FOS-related signaling pathways in the treatment of periodontitis. Odanacatib, a specific inhibitor of cathepsin K, has shown preliminary therapeutic promise in periodontitis models. Studies have demonstrated that odanacatib not only suppresses inflammation and alveolar bone loss associated with periodontal disease (94) but also, when combined with tetracycline, promotes macrophage polarization toward an anti-inflammatory phenotype and enhances osteogenic capacity under inflammatory conditions, thereby improving bone density (95).On the other hand, LHVS, a broad-spectrum irreversible inhibitor, lacks direct evidence in the context of periodontitis. However, it has been reported to alleviate chronic inflammatory responses by inhibiting cathepsin S (96), indicating its potential to indirectly influence periodontal tissue homeostasis through modulation of immune cell function. Although no studies have directly reported specific interactions between FOS and these compounds in periodontitis, molecular docking has demonstrated their favorable binding characteristics with the FOS protein. This provides new insights into their potential mechanisms of action in periodontitis and offers a theoretical basis for developing multi-target therapeutic strategies aimed at the FOS signaling axis. Nevertheless, the specific biological effects of these interactions require further experimental validation. Second, scRNA-seq identified 14 distinct immune cell populations, with T lymphocytes emerging as central regulators of periodontal inflammation. Dynamic alterations in T helper cell subsets (Th1, Th2, Th17, Treg) were observed during disease progression, consistent with previous research. Notably, activated T cells promoted osteoclastogenesis through IL-17 secretion and RANKL activation *via* the secreted osteoclastogenic factor (SOFAT) (97). These findings align with earlier demonstrations of T cell-dependent osteoclast differentiation in patients with PD (98). The immunopathological role of T cells involves complex interactions with innate immune cells (neutrophils, macrophages) and modulation of B cell responses through cytokine networks (99). While contributing to tissue destruction, T cells may also play a role in reparative processes, with memory T cell populations potentially influencing disease recurrence (100). These dual functions highlight the necessity for targeted immunomodulation rather than broad immunosuppression.

Firstly, the current analysis relies exclusively on RNA-level data. Although potential biomarkers were screened through bioinformatic approaches and validated at the mRNA level, their expression and function at the protein level remain unconfirmed and require further verification. Secondly, the RT-qPCR validation was performed with a relatively small clinical sample size, limiting statistical power and potentially constraining the clinical applicability of the findings. Furthermore, while the discriminatory ability of the predictive model was assessed using the GEO dataset (GSE10334), all data were derived from public databases. The absence of external validation with an independent clinical cohort restricts the evaluation of the model’s generalizability across broader populations and introduces potential overfitting risks. At the mechanistic level, although ISR-related candidate biomarkers were identified, their specific interactive roles in the pathogenesis and progression of periodontitis remain unclear. Functional experiments are needed to validate the regulatory effects of these genes on key signaling pathways. Additionally, the molecular docking analysis focused solely on the FOS protein and a limited number of compounds. Experimental validation is required to confirm the actual binding between these compounds and FOS, as well as to assess their interventional effects in cellular models of periodontitis. To address these limitations, future research will focus on the following aspects: expanding the sample size of independent clinical cohorts while validating candidate biomarker expression at the protein level to comprehensively evaluate their diagnostic value; verifying the binding affinity between FOS and the predicted compounds through *in vitro* assays and evaluating their effects on periodontitis-related phenotypes in cellular models; and employing techniques such as dual-luciferase reporter assays and co-immunoprecipitation (Co-IP) to elucidate the interaction mechanisms between the biomarkers and core signaling pathways in periodontitis. These efforts will systematically enhance the scientific rigor and clinical translational potential of the study findings.

## 5 Conclusion

This study identified five biomarkers (BTG2, DERL3, FOS, HSPA13, and YOD1) associated with the ISR through comprehensive bioinformatics analyses, suggesting their potential for therapeutic applications in PD. Additionally, single-cell analysis highlighted the central role of T cells in PD, offering valuable insights for further investigation into their specific mechanisms within the disease. While this research provides new bioinformatics perspectives and evidence for PD-related studies, further validation of these biomarkers in a broader range of clinical samples, as well as the use of advanced research tools such as animal models, is necessary to strengthen their reliability. Moreover, ongoing research into BTG2, DERL3, FOS, HSPA13, YOD1, and T cells in the context of PD will continue to deepen our understanding of their roles in the disease.

## Data Availability

The datasets ANALYZED for this study can be found in the[Gene Expression Omnibus (GEO) database] [http://www.ncbi.nlm.nih.gov/geo/, GSE16134, GSE10334, and GSE171213].
