# Supplementary figures and images for: Integrative bulk and single-cell transcriptome analyses reveal integrated stress response-related biomarkers in periodontitis with experimental validation
Source: Front Immunol. 2025 Dec 11;16:1705047. doi: 10.3389/fimmu.2025.1705047 (PMC12739553; doi:10.3389/fimmu.2025.1705047)

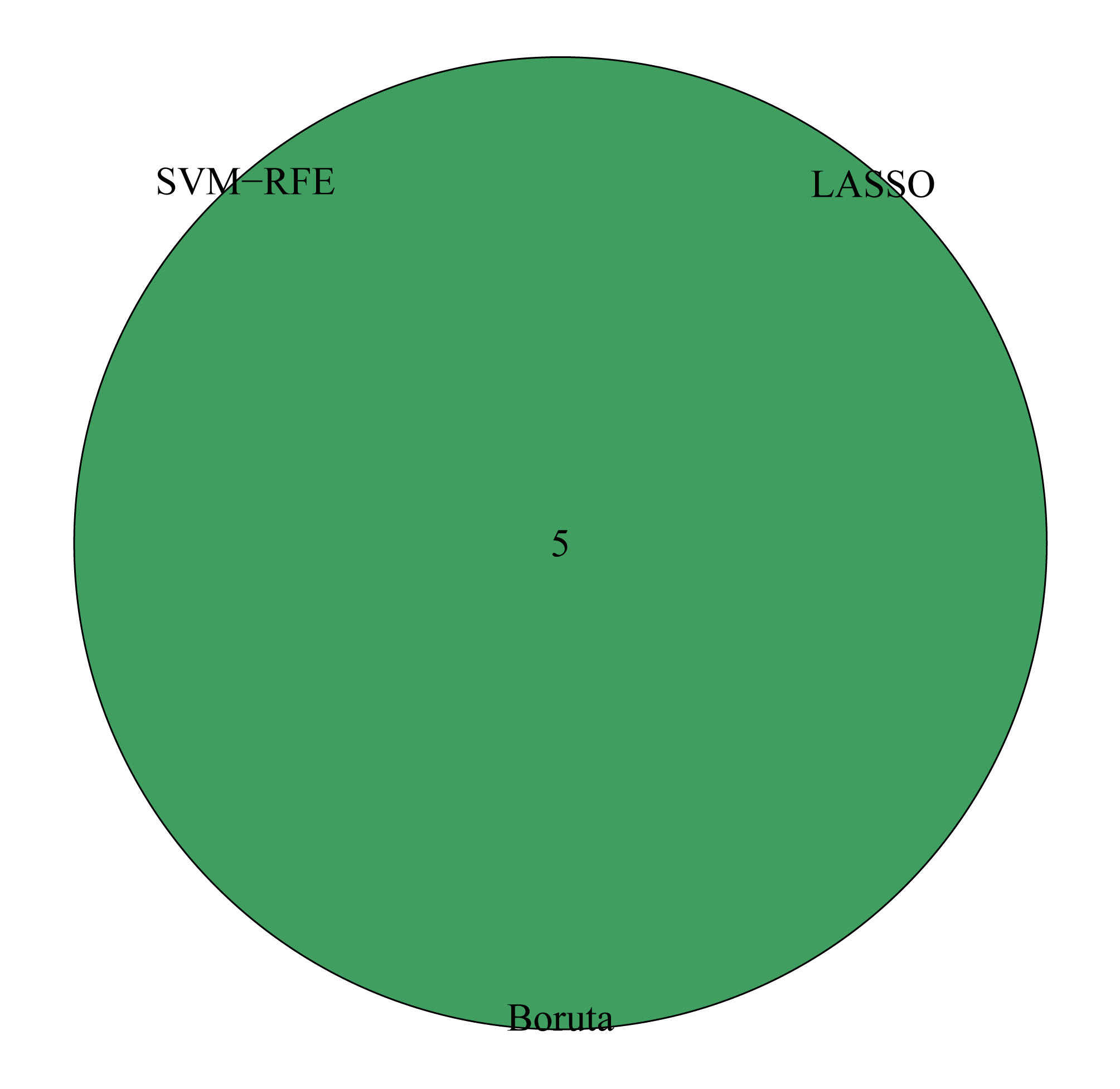

Supplement: Supplementary file 1 [file Image1.tif]

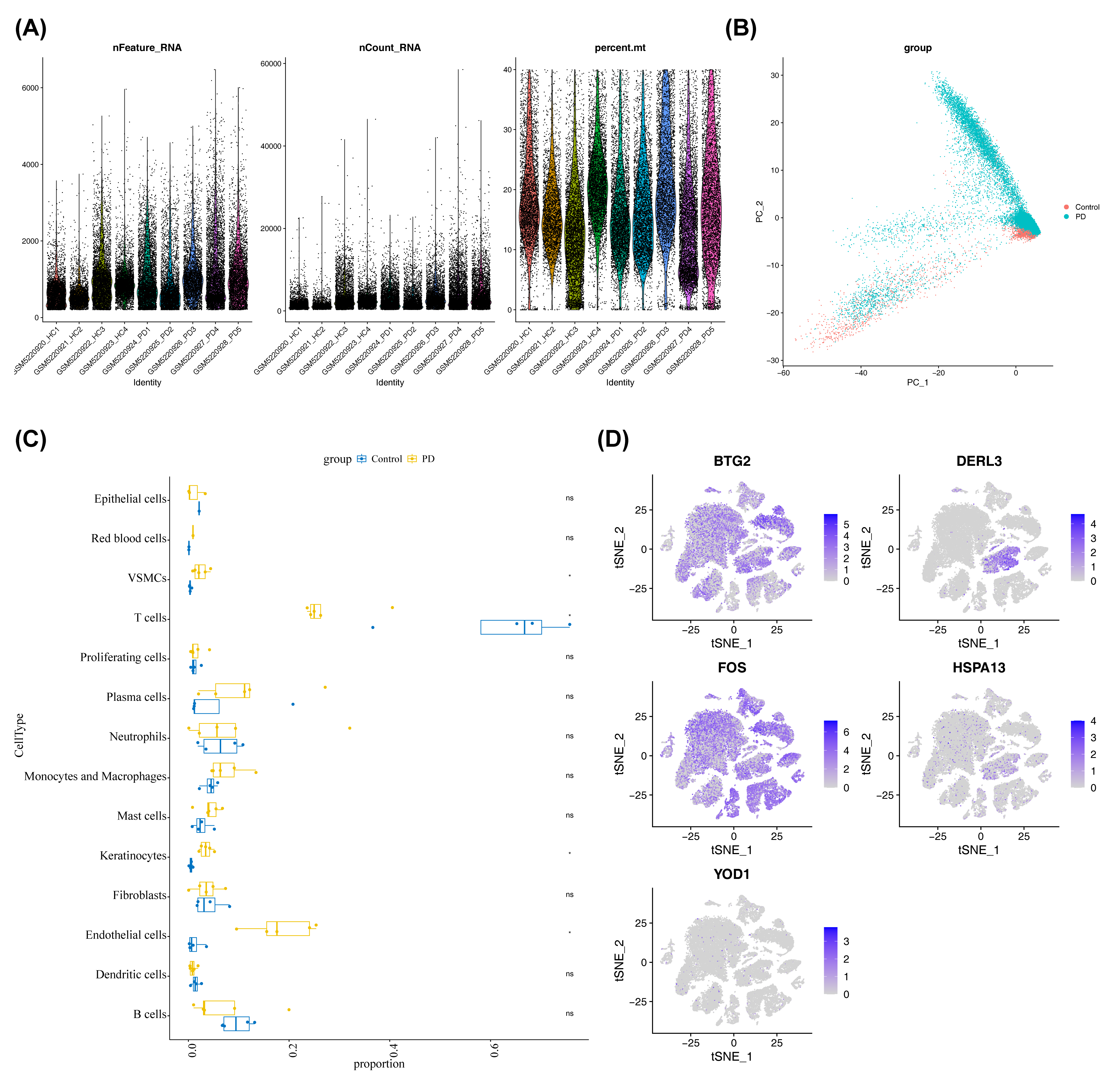

Supplement: Supplementary file 2 [file Image2.tif]
