# Supplementary material for: Integrative bulk and single-cell transcriptome analyses reveal integrated stress response-related biomarkers in periodontitis with experimental validation
Source: Front Immunol. 2025 Dec 11;16:1705047. doi: 10.3389/fimmu.2025.1705047 (PMC12739553; doi:10.3389/fimmu.2025.1705047)
Supplement: Supplementary file 15 [file Table13.docx]

Supplementary Material

# Supplementary Figures and Tables

## Supplementary Figures


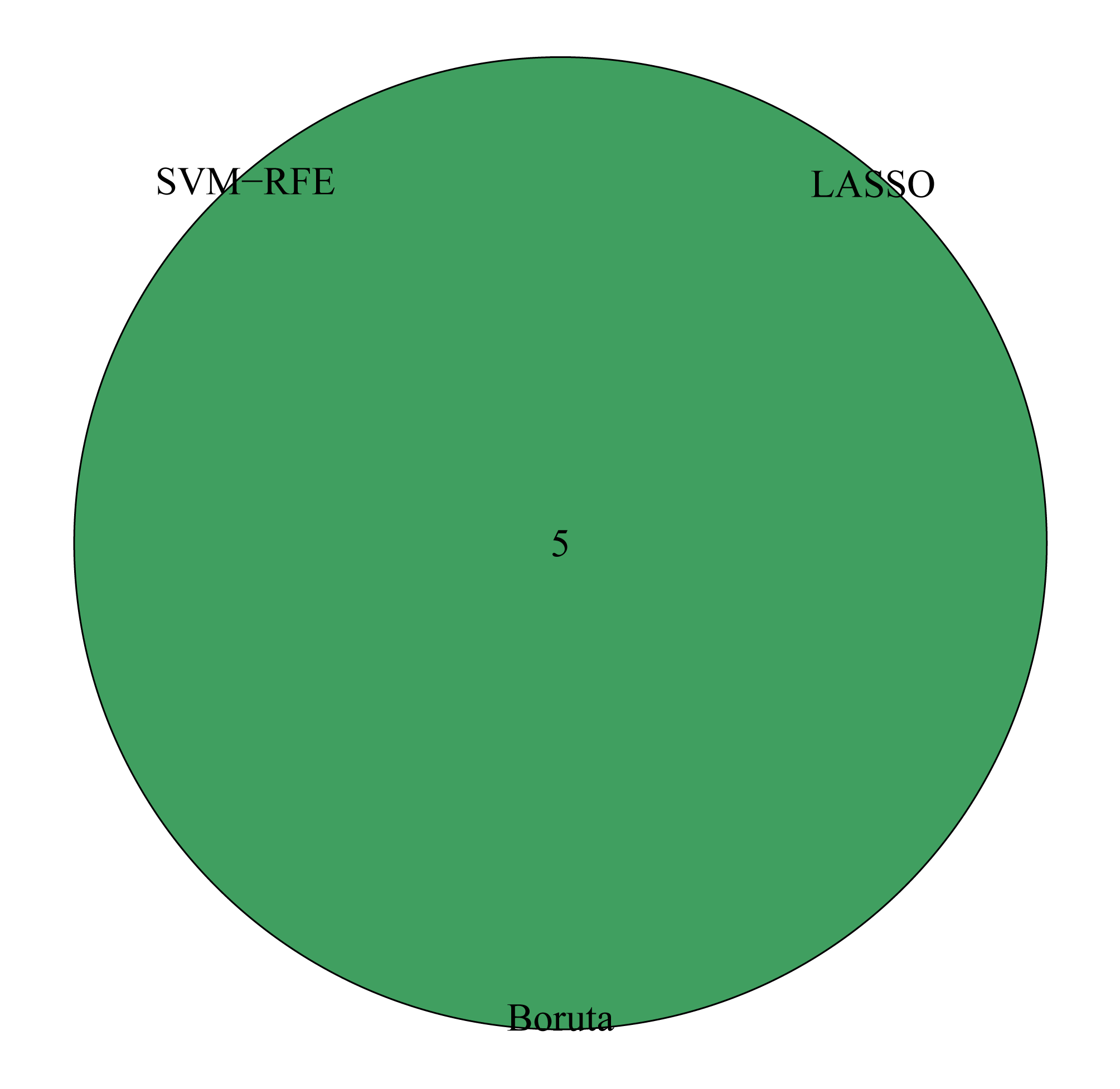


**Fig. S1. The Venn diagram illustrated the overlap of signature genes identified by the three machine learning algorithms.**


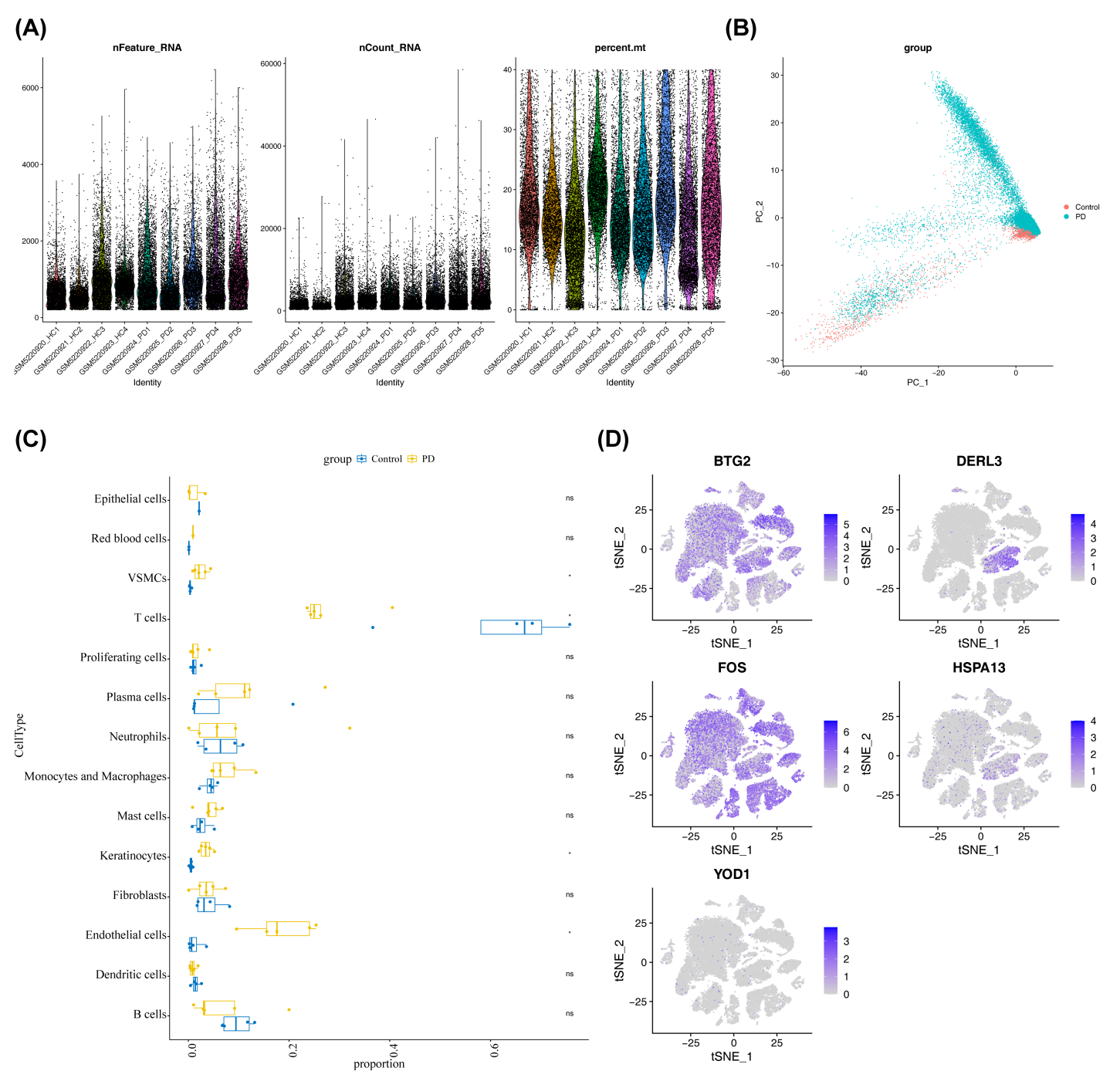


**Fig. S2. Preprocessing of single-cell analysis and distribution of biomarkers in annotated cells. (A)** Scatter plots after quality control and filtering of single-cell data. Each point represented a cell. The three subplots from left to right showed the number of genes per cell, the total RNA expression count, and the percentage of mitochondrial RNA, respectively. The x-axis indicated sample names, and the y-axis represented the quantity or proportion. **(B)** Sample distribution after PCA dimensionality reduction. Each point represented a cell. The x-axis and y-axis corresponded to the first two dimensions after PCA, and different colors distinguished the two sample types. Closer points indicated higher similarity between cells. **(C)** Proportional differences of PD and control samples across different cell types. Each point represented cells in a sample. The y-axis indicated cell types, and the x-axis represented the cell proportion. Yellow denoted PD samples, and blue denoted control samples. **(D)** Expression of biomarkers in t-SNE visualization. Each point represented a cell. Colors indicated gene expression levels, with darker blue representing higher expression.


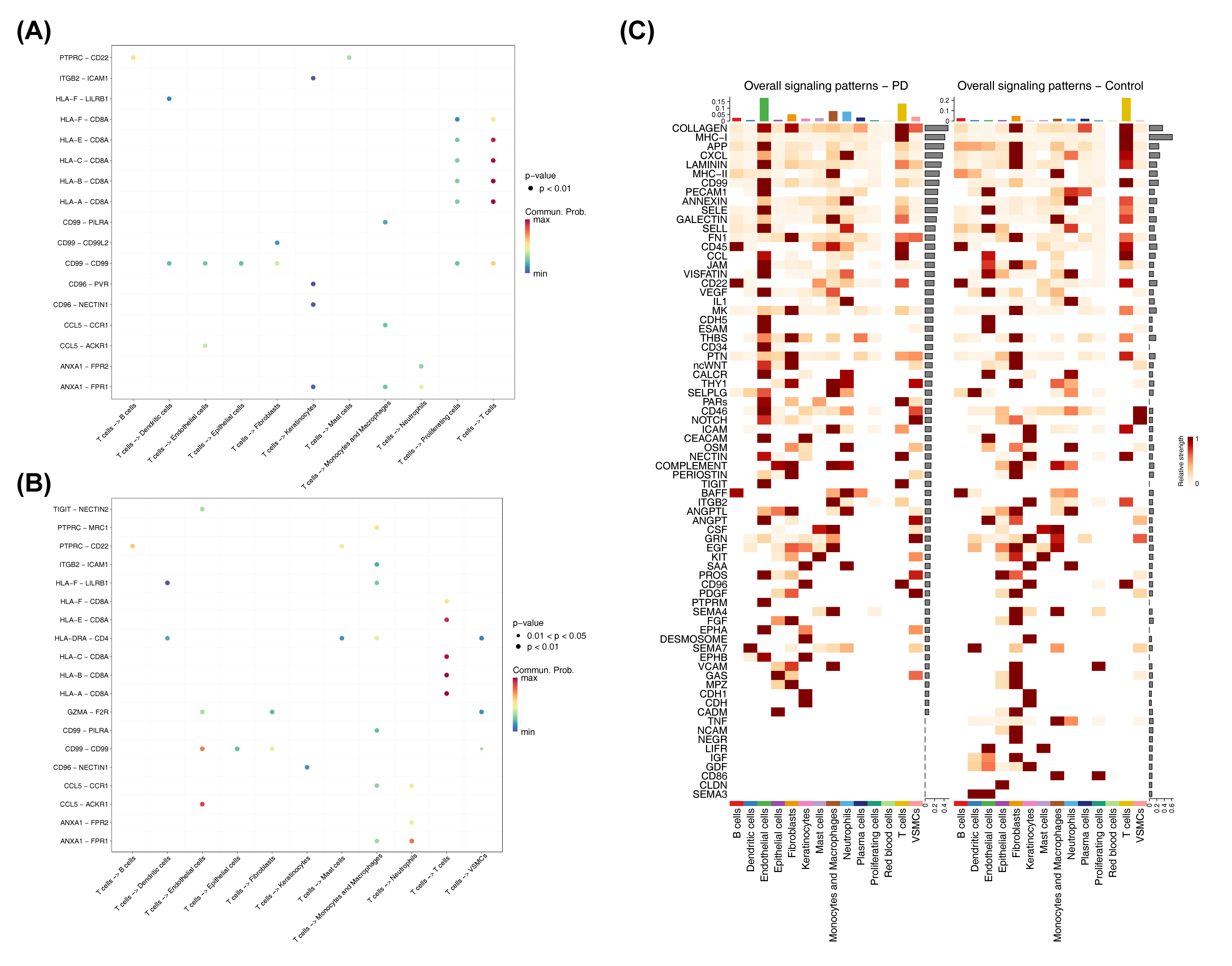


**Fig. S3. Cell communication results. (A-B)** Cell communication outcomes in T cells between PD and control samples: **(A)** control samples, **(B)** PD samples. **(C)** Overall signaling pattern comparison. In the upper panel, the x-axis represented cell types and the y-axis represented pathways. The left heatmap showed the overall signal strength in PD samples, and the right heatmap showed that in control samples.


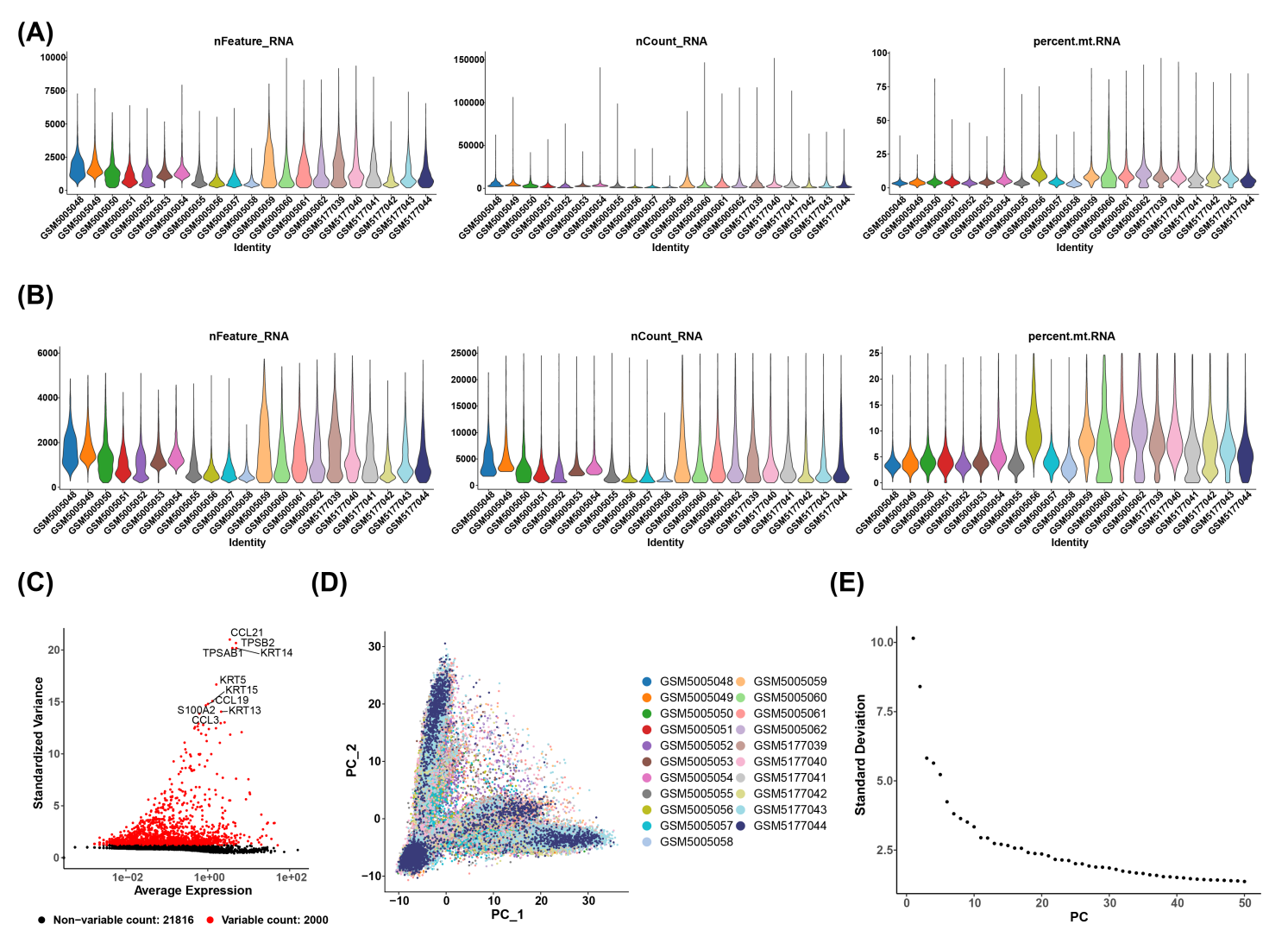


**Figure S4. Preprocessing of single-cell data from the GSE164241 dataset. (A-B)** Scatter plots of the single-cell data after quality control and filtering. Each point represents a single cell. The three subplots from left to right show: the number of genes per cell, the total RNA expression per cell, and the percentage of mitochondrial RNA. The x-axis indicates the sample names, and the y-axis indicates the values or percentages. **(A)** Before quality control. **(B)** After quality control. **(C)** Scatter plot of highly variable genes. Each point represents a gene. The x-axis indicates the average expression, and the y-axis indicates the standard deviation. **(D)** Scatter plot of principal component analysis. **(E)** Scree plot of principal component analysis. The x-axis indicates the principal component number, and the y-axis indicates the standard deviation.


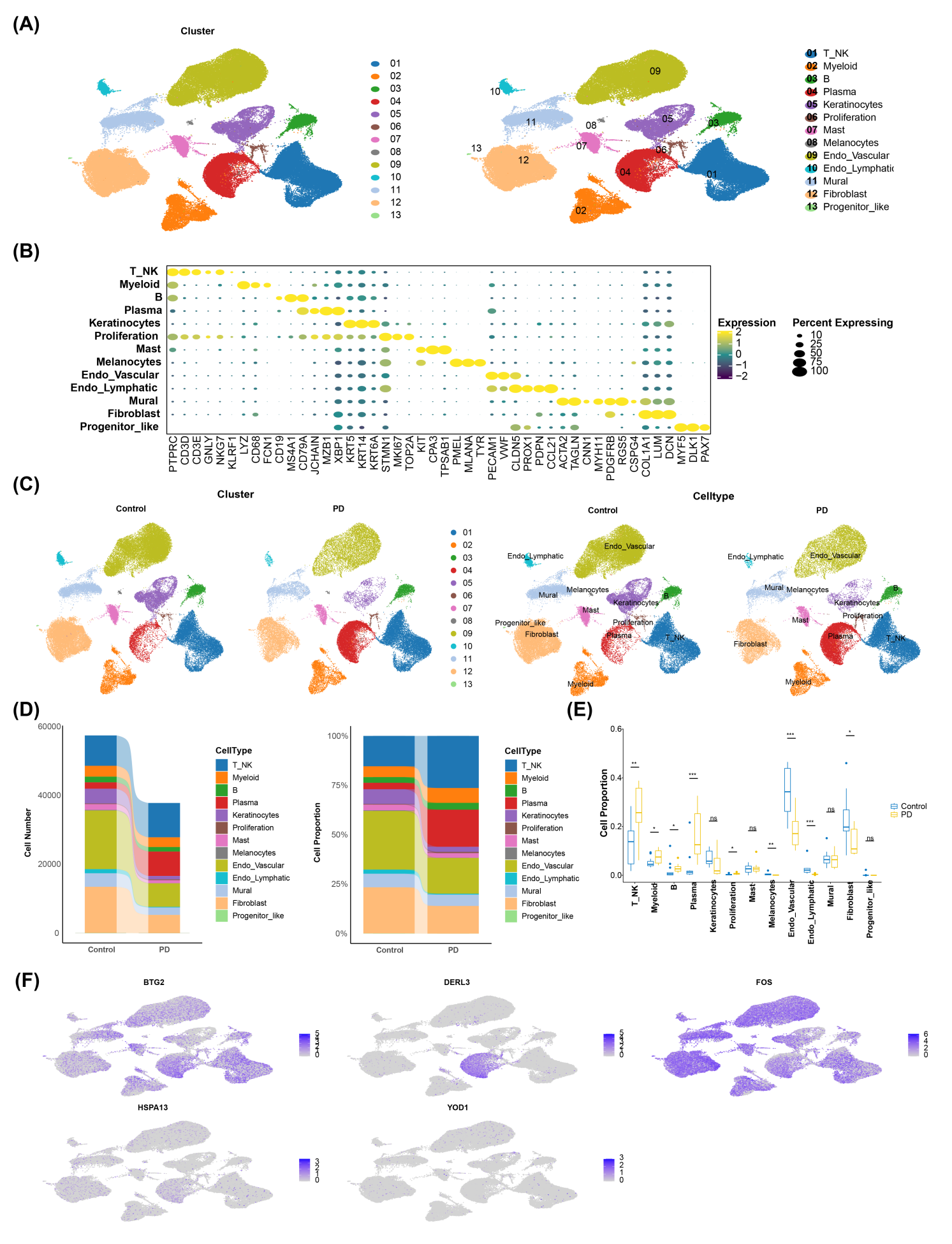


**Figure S5. Clustering of the GSE164241 single-cell data and biomarker expression across cell types. (A)** Visualization of cell cluster annotations. Cells were colored according to their assigned cluster in the UMAP plot, with closer colors indicating higher similarity. **(B)** Bubble plot depicting the expression of canonical cell type markers. The x-axis shows representative markers, and the y-axis shows the cell types. **(C)** Distribution of cell types between the periodontitis and control groups. **(D)** Stacked bar chart showing the proportional abundance of each cell type within each group. **(E)** Bar chart showing the differences in cell type proportions between groups. Blue bars represent the control group, and yellow bars represent the disease group. *: p < 0.05, **: p < 0.01, ***: p < 0.001, ns: not significant. **(F)** Dot plot showing the expression distribution of the identified biomarkers across different cell types. Intensity of the purple color indicates the expression level.

## Supplementary Tables

**Table S1 List of ISR-Related Genes**

**Table S2 Primer Sequence Table**

**Table S3 Thermal Cycling Conditions for RT-qPCR**

**Table S4 Specific genetic information corresponding to the three regions of the Venn Diagram respectively**

**Table S5 GO enrichment analysis results**

**Table S6 KEGG enrichment analysis results**

**Table S7 The results of correlation analysis among different immune cells**

**Table S8 The results of the correlation analysis between immune cells and biomarkers**

**Table S9 Drug Prediction Results**

**Table S10 Molecular Docking Results**

**Table S11 Specific marker genes related to each annotated cell type**

**Table S12 RNA concentrations of all samples**
